# Transcriptional responses of the nerve agent-sensitive brain regions amygdala, hippocampus, piriform cortex, septum, and thalamus following exposure to the organophosphonate anticholinesterase sarin

**DOI:** 10.1186/1742-2094-8-84

**Published:** 2011-07-21

**Authors:** Kimberly D Spradling, Lucille A Lumley, Christopher L Robison, James L Meyerhoff, James F Dillman

**Affiliations:** 1 Cell and Molecular Biology Branch, US Army Medical Research Institute of Chemical Defense (USAMRICD), 3100 Ricketts Point Road, Aberdeen Proving Ground, MD 21010-5400, USA; 2 Neurobehavioral Toxicology Branch, US Army Medical Research Institute of Chemical Defense (USAMRICD), 3100 Ricketts Point Road, Aberdeen Proving Ground, MD 21010-5400, USA; 3 US Army Center for Environmental Health Research, 568 Doughten Drive, Fort Detrick, MD 21702-5010, USA

**Keywords:** Nerve Agent, Chemical Warfare, Organophosphate, Sarin, Seizure, Neuroinflammation, Cytokine, Chemokine, Microarray, Transcriptomics

## Abstract

**Background:**

Although the acute toxicity of organophosphorus nerve agents is known to result from acetylcholinesterase inhibition, the molecular mechanisms involved in the development of neuropathology following nerve agent-induced seizure are not well understood. To help determine these pathways, we previously used microarray analysis to identify gene expression changes in the rat piriform cortex, a region of the rat brain sensitive to nerve agent exposure, over a 24-h time period following sarin-induced seizure. We found significant differences in gene expression profiles and identified secondary responses that potentially lead to brain injury and cell death. To advance our understanding of the molecular mechanisms involved in sarin-induced toxicity, we analyzed gene expression changes in four other areas of the rat brain known to be affected by nerve agent-induced seizure (amygdala, hippocampus, septum, and thalamus).

**Methods:**

We compared the transcriptional response of these four brain regions to sarin-induced seizure with the response previously characterized in the piriform cortex. In this study, rats were challenged with 1.0 × LD_50 _sarin and subsequently treated with atropine sulfate, 2-pyridine aldoxime methylchloride, and diazepam. The four brain regions were collected at 0.25, 1, 3, 6, and 24 h after seizure onset, and total RNA was processed for microarray analysis.

**Results:**

Principal component analysis identified brain region and time following seizure onset as major sources of variability within the dataset. Analysis of variance identified genes significantly changed following sarin-induced seizure, and gene ontology analysis identified biological pathways, functions, and networks of genes significantly affected by sarin-induced seizure over the 24-h time course. Many of the molecular functions and pathways identified as being most significant across all of the brain regions were indicative of an inflammatory response. There were also a number of molecular responses that were unique for each brain region, with the thalamus having the most distinct response to nerve agent-induced seizure.

**Conclusions:**

Identifying the molecular mechanisms involved in sarin-induced neurotoxicity in these sensitive brain regions will facilitate the development of novel therapeutics that can potentially provide broad-spectrum protection in five areas of the central nervous system known to be damaged by nerve agent-induced seizure.

## Background

Organophosphorus (OP) nerve agents, such as sarin (O-isopropyl methylphosphonofluoridate), irreversibly inhibit the enzyme acetylcholinesterase (AChE). This inactivation of AChE causes a toxic accumulation of the neurotransmitter acetylcholine (ACh) and results in over-stimulation of muscarinic and nicotinic ACh receptors [1-3]. Due to the continuous stimulation of muscles, glands, and central nervous system, victims exposed to these poisonous agents develop myosis, tightening of the chest, difficulty breathing, and a general loss of bodily functions. As symptoms progress, the victims suffer from convulsive spasms and seizures that can quickly progress to status epilepticus (SE), which has been strongly associated with brain damage in survivors, and death [1,3-6].

Current medical countermeasures against toxic levels of nerve agents can increase survival if administered within a short period of time following exposure, but they may not fully prevent neuropathology or functional impairment [3,6-9]. Although these countermeasures are readily available to soldiers in a combat setting, they are not accessible to the general public in case of a terrorist attack. The anticipated response time to treat civilian casualties exposed to nerve agent is estimated to be at least 30 min [6], and many will have already initiated seizures by the time medical personnel arrive. Rapidly terminating nerve agent-induced seizures is critical because their duration and intensity have been directly linked to brain damage following exposure [4,10-12]. Previous studies have shown signs of neuropathology present within 20 min of seizure onset and have demonstrated the increased difficulty in terminating seizures lasting beyond 40 min [6,10]. Therefore, it is important to understand the mechanism of nerve agent-induced brain injury and identify treatments that are effective when administered after the initiation of seizures and the secondary responses that lead to brain injury.

We previously characterized the transcriptional response of rat piriform cortex following sarin exposure [51]. We found that critical gene expression profile differences correlated with seizure induction and identified secondary responses that potentially lead to brain injury and cell death. In addition to the piriform cortex, other brain regions have been identified as sensitive to varying degrees to nerve agent exposure. These include the amygdala, hippocampus, septum, and thalamus [3,6,10,13]. To understand in greater detail the molecular responses of these brain regions to nerve agent, we utilized oligonucleotide microarrays to define the temporal transcriptional responses of these brain regions following sarin-induced seizure in a rat model. We then compared the transcriptional profiles of these four brain regions to the transcriptional response previously characterized in the piriform cortex to identify the common and unique molecular mechanisms significantly affected by sarin-induced seizure in these five sensitive brain regions.

## Methods

### Sarin exposure

Male Sprague-Dawley rats (350-500 g) were obtained from Charles River Laboratories (Wilmington, MA). They were housed in a temperature-controlled room with a 12-h light/12-h dark cycle and given food and water *ad libitum*. The research for this study was conducted at the United States Army Medical Research Institute of Chemical Defense (USAMRICD; Aberdeen Proving Ground, MD), which is fully accredited by the Association for Assessment and Accreditation of Laboratory Animal Care, International. All of the animal procedures were approved by the Institute Animal Care and Use Committee at USAMRICD and conducted in accordance with the principles stated in the *Guide for the Care and Use of Laboratory Animals *(National Research Council, 1996) and the Animal Welfare Act of 1966 (P.L. 89-544), as amended.

PhysioTel^® ^F40-EET transmitters (Data Sciences International, St. Paul, MN) were surgically implanted into the animals to record bi-hemispheric cortical electroencephalogram (EEG) activity, body temperature, and gross motor activity throughout the study. After a two-week recovery period, the animals were challenged with 1 × LD_50 _sarin (108 μg/kg, sc) that was obtained and diluted in sterile saline at USAMRICD. One minute after seizure onset, animals were treated with atropine sulfate (2 mg/kg; Sigma-Aldrich, St. Louis, MO) and 2-pyridine aldoxime methylchloride (2-PAM; 25 mg/kg; Sigma-Aldrich), both administered in a single injection (im). Thirty minutes later, animals used for the 1-h to 24-h time points were given the anticonvulsant diazepam (10 mg/kg, sc; TW Medical Veterinary Supply, Austin, TX). Control animals received an equivalent volume of vehicle (saline), atropine sulfate, 2-PAM, and diazepam. Naïve animals received no injections.

Behavioral observations were documented for each animal following exposure and placed in one of three categories (mild, moderate, or severe). The total was then calculated and graphed using the total number of toxic signs listed in the moderate (e.g., loss of posture, excessive salivation and/or lacrimation, and body tremors) and severe (e.g., complete loss of posture, clonic-tonic convulsions, and gasping) categories. These behavioral observations corresponded with the five stages of behavioral seizure intensity, which were rated using a modified Racine scale score [14]: stage 0 = baseline behaviors, including resting, grooming, chewing, and sleeping; stage 1 = inactivity, unusual posture, piloerection, frozen posture, clumsy motion, and excessive grooming or chewing; stage 2 = oral tonus, head bobs, and body tremors; stage 3 = forelimb myoclonus, prostrate body extension, and salivation or lacrimation; stage 4 = loss of posture, whole body tremors, rigidity, body jerks, and forelimb myoclonus followed by rearing; and stage 5 = complete loss of posture, falling or generalized tonic-clonic convulsions, and gasping. Statistical significance between sarin-exposed seizing animals and their controls was calculated using Student's *t*-test.

Animals were euthanized by decapitation at 0.25, 1, 3, 6, and 24 h after seizure onset. The amygdala, hippocampus, septum, and thalamus were immediately collected from each animal at the appropriate time point. Three animals were used for each experimental group (naïve, saline control, and sarin-exposed seizure) at each time point, with the exception of 1-h saline control, 3-h sarin-exposed seizure, and 24-h sarin-exposed seizure (n = 4). Each tissue was immediately snap-frozen in liquid nitrogen and stored at -80°C until use.

### Sample preparation for microarray hybridization

Brain tissues were homogenized in RNeasy lysis buffer (QIAGEN, Valencia, CA) at three intervals of 30 sec each using the Mini-Beadbeater-96 (Biospec Products, Bartlesville, OK) and 6.35 mm stainless steel beads. Each homogenate was subsequently centrifuged for 10 min at 16,110 × g at room temperature, and the supernatant was transferred to a new microcentrifuge tube. Total RNA was then extracted and DNase I-treated using the RNeasy Mini Kit and RNase-Free DNase Set (QIAGEN) according to the manufacturer's protocol. The quantity and quality of the RNA was determined with a NanoDrop ND-1000 UV-vis spectrophotometer (Thermo Scientific, Wilmington, DE) and an Agilent Bioanalyzer (Agilent Technologies, Santa Clara, CA) throughout sample processing. Total RNA was processed for hybridization to GeneChip^® ^Rat Genome 230 2.0 oligonucleotide arrays (Affymetrix, Inc., Santa Clara, CA) using the BioArray Single-Round RNA Amplification and Biotin Labeling System (Enzo Life Sciences, Inc., Farmingdale, NY) as previously described [15]. In brief, 1 μg (amygdala, hippocampus, and thalamus) or 500 ng (septum) of total RNA was used to generate first strand cDNA by using a T7-linked oligo(dT) primer. After second strand synthesis, *in vitro *transcription was performed with biotinylated UTP and CTP for cRNA amplification. Biotinylated target cRNA generated from each sample was processed according to the manufacturer's protocol using an Affymetrix GeneChip Instrument System http://affymetrix.com/support/ technical/manual/expression_manual.affx as previously described [15].

All microarray experiments were performed to comply with Minimal Information About a Microarray Experiment (MIAME) protocols and details can be found at the Gene Expression Omnibus (GEO) accessible through GEO Series accession number GSE28435. The data discussed in this publication have been deposited in the National Center for Biotechnology Information's Gene Expression Omnibus (GEO; http://www.ncbi.nlm.nih.gov/geo/) and are accessible through GEO

Series accession number GSE28435.

### Microarray data analysis

Raw signal intensities from each GeneChip^® ^were imported into Partek Genomics Suite v6.4 (Partek, Inc., St. Louis, MO) along with those from the piriform cortex samples [51]. The signal intensities were normalized using the robust multiarray averaging (RMA) algorithm [16]. Normalized data for all five brain regions (amygdala, hippocampus, piriform cortex, septum, and thalamus) were analyzed by principal component analysis (PCA) [17] to identify patterns in the dataset and highlight similarities and differences among the samples. The major sources of variability identified within the dataset were used as grouping variables for analysis of variance (ANOVA). The calculated *p*-value and geometric fold change for each probeset identifier were imported into Ingenuity Pathways Analysis (IPA; Ingenuity^® ^Systems, http://www.ingenuity.com) to identify the canonical pathways, biological functions, and networks of genes significantly affected by sarin-induced seizure. Biological functions are categories that genes are classified into based on their cellular or physiological role in a healthy or diseased organism. Genes may be classified into more than one biological function. A canonical pathway is a well-established signaling or metabolic pathway that is manually curated on the basis of published literature. Canonical pathways are fixed prior to data input and do not change upon data input. Networks are distinct from canonical pathways in that they are built *de novo *from input data based on known molecular interactions identified in the published scientific literature. To identify canonical pathways that were most significant to the dataset, molecules that met the designated *p*-value cutoff (≤ 0.05) and were associated with a canonical pathway in Ingenuity's Knowledge Base were considered for the analysis. The significance of the association between the dataset and the canonical pathway was measured in two ways: 1) A ratio of the number of molecules from the data set that mapped to the pathway divided by the total number of molecules that mapped to the canonical pathway was displayed. 2) Fisher's exact test was used to calculate a *p*-value determining the probability that the association between the genes in the dataset and the canonical pathway was explained by chance alone. To determine networks of genes significantly affected by sarin exposure, molecules were overlaid onto a global molecular network developed from information contained in Ingenuity's Knowledge Base. Networks of molecules were then algorithmically generated based on their connectivity. The Functional Analysis of a network identified the biological functions and/or diseases that were most significant to the molecules in the network. The network molecules associated with biological functions and/or diseases in Ingenuity's Knowledge Base were considered for the analysis. Right-tailed Fisher's exact test was used to calculate a *p*-value determining the probability that each biological function and/or disease assigned to that network is due to chance alone.

### Multiplexed RT-PCR

The GenomeLab Gene Expression Profiler (GeXP; Beckman Coulter, Inc., Brea, CA) genetic analysis system was used to measure the expression levels of 21 differentially expressed cytokines or chemokines (see Additional File 1) by multiplexed RT-PCR to validate the microarray data. Primers were designed using the eXpress Designer module of the GenomeLab eXpress Profiler software, with each primer consisting of 20 nucleotides of gene-specific sequence as well as a universal primer sequence. RT-PCR product sizes ranged from 151 to 351 nt with a 7-nt minimum separation size between each fragment (see Additional File 1). The custom multiplexed panel also contained glyceraldehyde 3-phosphate dehydrogenase (GAPDH) for normalization and an internal control gene (kanamycin resistance, Kan^r^).

RNA samples used in the microarray experiment and the GenomeLab GeXP Start Kit (Beckman Coulter, Inc.) were used for the RT-PCR reactions according to the manufacturer's protocol. The custom multiplex was first optimized by reverse primer dilution to attenuate the gene signals that were close to or above the linear detection limit of the GeXP system detector (130,000 RFU in raw data or 120,000 RFU in analyzed data) and to balance the signal of each peak within the multiplex reaction. The final concentrations of the reverse primers within the multiplex are shown in Additional File 2. Fifty nanograms of total RNA was reverse transcribed with the optimized reverse primer multiplex. Subsequently, 9.3 μl of cDNA from each RT reaction was transferred to the PCR reaction mix containing 20 nM of the forward primer set multiplex. All experiments included "no template" (i.e. without RNA) and "no enzyme" (i.e. without reverse transcriptase) negative controls to confirm the absence of peaks at the expected target sizes.

The fluorescently-labeled PCR products were diluted 1:20 in 10 mM Tris-HCl (pH 8), and 1 μl of each dilution was added to 38.5 μl sample loading solution along with 0.5 μl DNA size standard-400 (GenomeLab GeXP Start Kit). The GeXP system was then used to separate the amplified PCR products based on size by capillary gel electrophoresis and to measure their fluorescent dye signal strength in arbitrary units (A.U.) of optical fluorescence, which is the fluorescent signal minus background. The multiplexed RT-PCR data were initially analyzed using the Fragment Analysis module of the GenomeLab GeXP system software, followed by the eXpress Analysis module of the eXpress Profiler software. First, the length or size of the products was determined using the Fragment Analysis module. The fragment data, peak height, and peak area information was then imported into the analysis module of the eXpress Profiler software where the fragments were compared to the expected PCR product sizes to identify each transcript.

The expression of each gene within a sample was normalized to GAPDH expression to minimize inter-capillary variation, and the normalized intensity of each replicate (n ≥ 3) was used to calculate an average intensity of each sample group (i.e. control or sarin-induced seizure at each time point). The fold expression difference between control and sarin-induced seizure samples was then evaluated for all genes at each time point and compared to the fold expression changes obtained by microarray analysis.

## Results

### Clinical manifestations of sarin exposure

Male Sprague-Dawley rats were challenged with 1 × LD_50 _sarin or saline (as control) as described under *Materials and Methods*. EEG monitoring showed that seizures were induced in approximately 50% of the sarin-exposed animals, with a mean latency of 10.2 min. Behavioral seizure intensity was scored using a modified Racine scale [14]. The amount of moderate and severe toxic signs exhibited by sarin-exposed seizing animals was significantly greater (*p *< 0.0001) than their controls, with the control animals having an average toxic signs score of 0.08 and the seizing animals having an average score of 10.88 (Spradling et al., submitted).

### Transcriptional analysis reveals gene expression profile differences correlated with brain region and time following seizure onset

**Figure 1:**
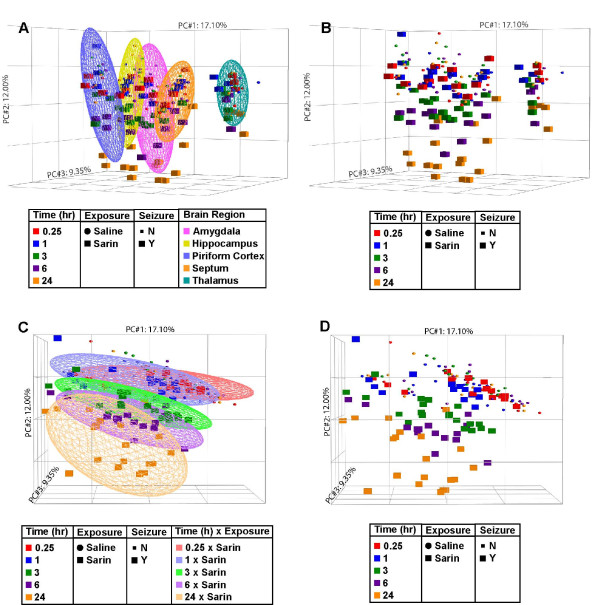
**Principal component analysis reveals sample partitioning based on brain region and time following seizure onset**. Rat amygdala, hippocampus, piriform cortex, septum, and thalamus were collected at the specified times after seizure onset and processed for oligonucleotide microarray analysis. The raw signal intensities were normalized using the RMA algorithm and visualized using PCA to identify major sources of variability in the data. Each point on the PCA represents the gene expression profile of an individual animal. Point shape corresponds to exposure condition, point color corresponds to the time after seizure onset at which the tissue was collected, and point size indicates absence or occurrence of sarin-induced seizure. Ellipsoids highlight partitioning of samples based on brain region (A) and time point following seizure onset at which the tissues were collected (C). The principal components in the three-dimensional graph represent the variability in gene expression levels seen within the dataset. Principal component 1 (PC#1, *x*-axis) accounts for 17.10% of the variability in the data; PC#2 (*y*-axis) represents 12.00% of the variability; and PC#3 (*z*-axis) represents 9.35% of the variability in gene expression levels seen within the dataset.

Total RNA was isolated from the amygdala, hippocampus, septum, and thalamus and processed for oligonucleotide microarray analysis. Raw data from these four brain regions and piriform cortex (Spradling et al., submitted) were normalized using the RMA algorithm [16] and analyzed by PCA (Figure 1) [17] to reduce the complexity of the multi-dimensional dataset. The resulting three-dimensional plot identified brain region type and time after seizure onset (0.25, 1, 3, 6, or 24 h) as major sources of variability within the dataset. Each point on the PCA represents the gene expression profile of an individual animal, and the distance between any two points is directly related to the similarity between those two samples. Therefore, samples that are near each other in the three-dimensional plot have a similar transcriptional profile while those that are further apart have dissimilar transcriptional profiles. The PCA highlights differences in gene expression from 0.25 h to 24 h following seizure onset. Ellipsoids reveal partitioning of samples based on brain region (Figure 1A, B) and time following seizure induction (Figure 1C, D). The amygdala, hippocampus, piriform cortex, and septum samples from sarin-exposed animals clearly partition away from controls and cluster together based on time after seizure onset, with the 24-h seizing animals separated the furthest from controls. This pattern is not seen as clearly in the thalamus samples. However, when the thalamus samples are viewed in the three-dimensional plot alone (excluding the other brain regions), this same pattern becomes more visible (data not shown).

### Canonical pathways associated with inflammation are significantly altered in sensitive brain regions of sarin-exposed seizing rats

The normalized microarray data was filtered based on brain region, and a two-way interaction ANOVA was performed to identify genes significantly altered based on exposure (saline or sarin) and time after seizure onset. The calculated *p*-values from each ANOVA were then imported into IPA to identify the canonical pathways most affected by sarin-induced seizure in each brain region over the 24-h time course. The top 800 genes that met the *p-*value cutoff (≤ 0.05) and were associated with a canonical pathway in the IPA Knowledge Base were considered for each analysis. Significant changes in gene expression were seen in all five brain regions following seizure occurrence, with the greatest effects in the piriform cortex (*p *value cutoff ≤ 1.650 × 10^-8^; Additional File 3), hippocampus (*p *≤ 1.75 × 10^-8^; Additional File 4), and amygdala (*p *≤ 2.800 × 10^-7^; Additional File 5). Fewer significantly altered genes were seen in the septum (*p *≤ 1.235 × 10^-5^; Additional File 6) and thalamus (*p *≤ 8.950 × 10^-5^; Additional File 7).

**Figure 2:**
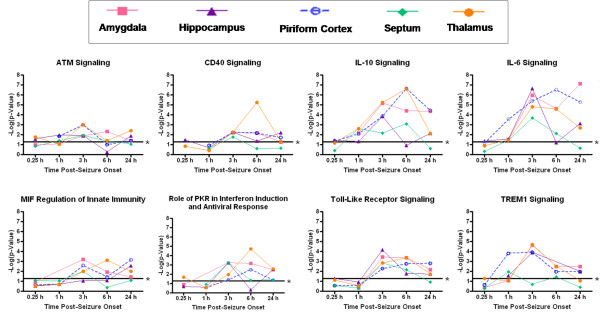
**Canonical pathways significantly altered in all five brain regions of sarin-exposed seizing animals**. A one-way ANOVA was performed to identify genes significantly changed in each brain region at each time point based on exposure (sarin vs. saline). The *p*-value and geometric fold change for each probeset ID were imported into IPA to identify the biological functions and canonical pathways most significantly affected by sarin-induced seizure at each time point. The top 800 genes that met the *p-*value cutoff (≤ 0.05) and were associated with a canonical pathway in the IPA Knowledge Base were considered for the analysis. The significance of the association between the dataset and the canonical pathway was calculated using Fisher's exact test. The -log of the *p*-value is graphed for each time point, with a threshold of 0.05 (or 1.3 when expressed as -log(*p*-value)) marked by an asterisk. The range of the y-axis was formatted the same to facilitate comparison across all the graphs in the figure.

Gene ontology analysis revealed numerous canonical pathways that were significantly altered by sarin-induced seizure in all five brain regions. Those that were significantly affected in at least one brain region are shown in an additional table (see Additional File 8). Pathways highlighted in pink indicate those that were significantly affected by nerve agent-induced seizure in the corresponding brain region but not across all five regions examined, grey indicates pathways detected but not significant, and white indicates pathways not detected in the analyses. Some canonical pathways associated with an inflammatory response were significant across all five brain regions. These pathways are highlighted in red and include: ataxia telangiectasia mutated protein (ATM) signaling, CD40 signaling, interleukin (IL)-10 signaling, IL-6 signaling, macrophage migration inhibitory factor (MIF) regulation of innate immunity, role of double-stranded RNA-activated protein kinase (PKR) in interferon induction and antiviral response, toll-like receptor signaling, and triggering receptor expressed on myeloid cells 1 (TREM1) signaling. To analyze these pathways over the 24-h time course, each brain region dataset was filtered based on time. A one-way ANOVA was then performed to identify genes significantly changed in each brain region at each time point based on exposure (sarin vs saline). As detailed above, the *p*-value and geometric fold change for each probeset ID were imported into IPA, and the top 800 genes were considered for each analysis. The significance of the association between the dataset and the canonical pathway was calculated using Fisher's exact test, and the -log of the *p*-value was graphed for each time point to show the pathway alterations over time in each brain region (Figure 2).

**Figure 3:**
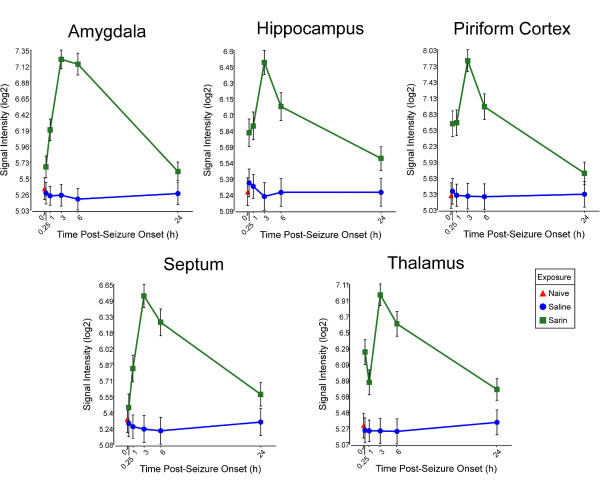
**Sarin-induced seizure up-regulates the expression of TNF-α in all five brain regions examined**. Brain tissues were collected at 0.25, 1, 3, 6, and 24 h after seizure onset and processed for oligonucleotide microarray analysis. TNF-α expression was induced as early as 0.25 h following seizure onset, peaked at 3 h, and returned to near control levels by 24 h after seizure onset.

**Figure 4:**
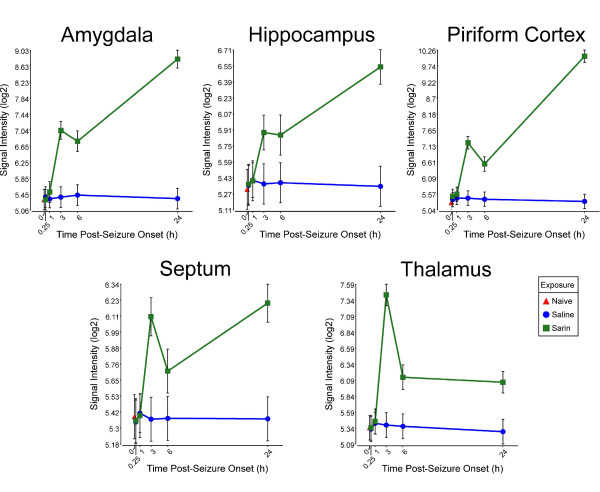
**Sarin-induced seizure up-regulates the expression of IL-6 in all five brain regions examined**. Tissues were collected at 0.25, 1, 3, 6, and 24 h after seizure onset and processed for oligonucleotide microarray analysis. IL-6 expression peaked at 3 h and decreased at 6 h in all brain regions. Expression then increased at 24 h after seizure onset in the amygdala, hippocampus, piriform cortex, and septum. IL-6 expression appeared to level off after 6 h in the thalamus.

**Figure 5:**
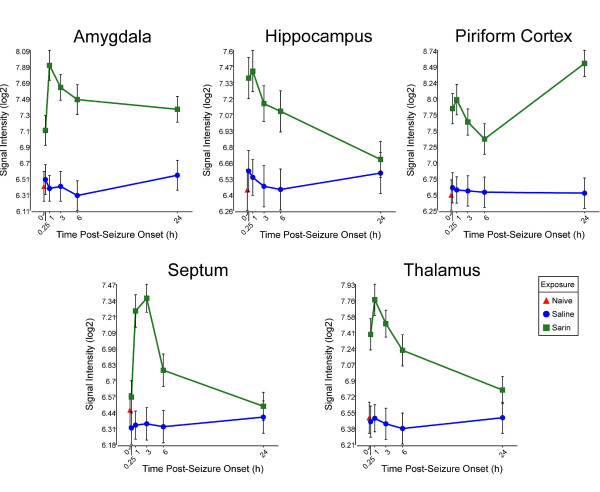
**Sarin-induced seizure up-regulates the expression of IL-1β in all five brain regions examined**. Tissues were collected at 0.25, 1, 3, 6, and 24 h after seizure onset and processed for oligonucleotide microarray analysis. IL-1β expression peaked at 1 h in the amygdala, hippocampus, and thalamus, while it peaked at 3 h in the septum and at 24 h in the piriform cortex. The expression levels decreased nearer to control level in the hippocampus, septum, and thalamus, while it remained approximately the same in the amygdala. However, IL-1β transcript level was increasing in the piriform cortex at our latest time point of 24 h.

Pro-inflammatory cytokines appear in many of the significantly altered pathways; therefore, we examined the transcriptional profiles of these significantly altered genes. We found that sarin-induced seizure up-regulates the expression of tumor necrosis factor-α (TNF-α) in all five brain regions examined. TNF-α expression is induced as early as 0.25 h after seizure onset, peaks at 3 h, and returns to near control levels at 24 h (Figure 3). IL-6 is also up-regulated in all five brain regions. However, IL-6 expression peaks at 3 h and drops at 6 h in all brain regions. Expression then increases at 24 h following seizure onset in all regions except the thalamus (Figure 4). Sarin-induced seizure also up-regulates the expression of IL-1β in all five brain regions examined. During the 24-h time course, IL-1β expression peaks at 1 h in the amygdala, hippocampus, and thalamus, while it peaks at 3 h in the septum and at 24 h in the piriform cortex. Expression returns to near control levels in the hippocampus, septum, and thalamus, while it stays nearly the same in the amygdala. IL-1β transcript level is increasing in the piriform cortex at our latest time point of 24 h (Figure 5).

We identified significantly altered canonical pathways unique for each brain region in sarin-exposed seizing animals. To further characterize these pathways, we filtered the data based on time, and an ANOVA was performed to identify genes significantly altered based on exposure (saline or sarin) at each time point following seizure onset. The unique pathways identified in the amygdala were apoptosis signaling; induction of apoptosis by HIV1; karatan sulfate biosynthesis; and role of osteoblasts, osteoclasts and chondrocytes in rheumatoid arthritis (Additional File 9). When the data was analyzed at individual time points, we found that apoptosis signaling was significant at 6 h; induction of apoptosis by HIV1 was significant at 3 and 6 h; karatan sulfate biosynthesis was significant at 0.25, 3, and 6 h; and role of osteoblasts, osteoclasts, and chondrocytes in rheumatoid arthritis was not significant at any of the time points analyzed. The unique pathways identified in the hippocampus were acute myeloid leukemia signaling; hypoxia signaling in the cardiovascular system; neuroprotective role of thimet oligopeptidase 1 (THOP1) in Alzheimer's disease; retinoic acid mediated apoptosis signaling; thyroid cancer signaling; and urea cycle and metabolism of amino groups (Additional File 10). When the data were analyzed at individual time points, we found that acute myeloid leukemia signaling and thyroid cancer signaling were significant at 3 h; urea cycle and metabolism of amino groups was significant at 24 h; and hypoxia signaling in the cardiovascular system was significant at 1, 3, and 24 h after seizure onset. Neuroprotective role of THOP1 in Alzheimer's disease and retinoic acid mediated apoptosis signaling were not significant at any of the individual time points examined. For the piriform cortex, unique pathways included extracellular receptor kinase/mitogen-activated protein kinase (ERK/MAPK) signaling; fibroblast growth factor (FGF) signaling; G-protein coupled receptor signaling; glioma invasiveness signaling; glycerophospholipid metabolism; IL-1 signaling; neuropathic pain signaling in dorsal horn neurons; peroxisome proliferator-activated receptor-α/retinoid × receptor-α (PPARα/RXRα) activation; and taurine and hypotaurine metabolism (Additional File 11). When analyzed at individual time points, we found that G-protein coupled receptor signaling and PPARα/RXRα activation were significant at 1 h; glioma invasiveness signaling and taurine and hypotaurine metabolism were significant at 24 h; ERK/MAPK signaling was significant at 1 and 24 h; and IL-1 signaling was significant at 3 and 6 h. FGF signaling, glycerophospholipid metabolism, and neuropathic pain signaling in dorsal horn neurons were not significant at any time point analyzed. The only unique pathway identified in the septum was human embryonic stem cell pluripotency (Additional File 12), which was significant only at 24 h when analyzed by individual time points. The largest number of unique pathways was identified in the thalamus. The 24 canonical pathways identified as being significant only in the thalamus after seizure onset were activation of interferon-regulatory factor (IRF) by cytosolic pattern recognition receptors; aryl hydrocarbon receptor signaling; autoimmune thyroid disease signaling; B cell receptor signaling; chronic myeloid leukemia signaling; colorectal cancer metastasis signaling; complement system; crosstalk between dendritic cells and natural killer cells; eicosanoid signaling; estrogen-dependent breast cancer signaling; glioblastoma multiforme signaling; glycosphingolipid biosynthesis-neolactoseries; granulocyte-macrophage colony-stimulating factor (GM-CSF) signaling; iCOS-iCOSL signaling in T helper cells; IL-8 signaling; molecular mechanisms of cancer; nuclear factor kappa-light-chain-enhancer of activated B cells (NF-κB) signaling; p53 signaling; platelet-derived growth factor (PDGF) signaling; phospholipase C signaling; primary immunodeficiency signaling; renal cell carcinoma signaling; role of nuclear factor of activated T cells (NFAT) in regulation of the immune response; and vitamin D receptor/retinoic acid × receptor (VDR/RXR) activation (Additional File 13). When the data were analyzed at individual time points, we found that primary immunodeficiency signaling was significant at 6 h; autoimmune thyroid disease, glioblastoma multiforme signaling, iCOS-iCOSL signaling in T helper cells, and phospholipase C signaling were significant at 24 h; estrogen-dependent breast cancer signaling was significant at 0.25 and 3 h; aryl hydrocarbon receptor signaling was significant at 3 and 6 h; B cell receptor signaling was significant at 0.25, 6, and 24 h; PDGF signaling was significant at 1, 3, and 6 h; VDR/RXR activation was significant at 1, 6, and 24 h; crosstalk between dendritic cells and natural killer cells was significant at 3, 6, and 24 h; p53 signaling was significant at 0.25, 3, 6, and 24 h; and molecular mechanisms of cancer was significant at 1, 3, 6, and 24 h following seizure onset. The remaining pathways were not significantly altered in the thalamus at any individual time point following sarin-induced seizure onset.

### Canonical pathways and networks of genes significantly altered across all brain regions from sarin-exposed seizing animals

**Figure 6:**
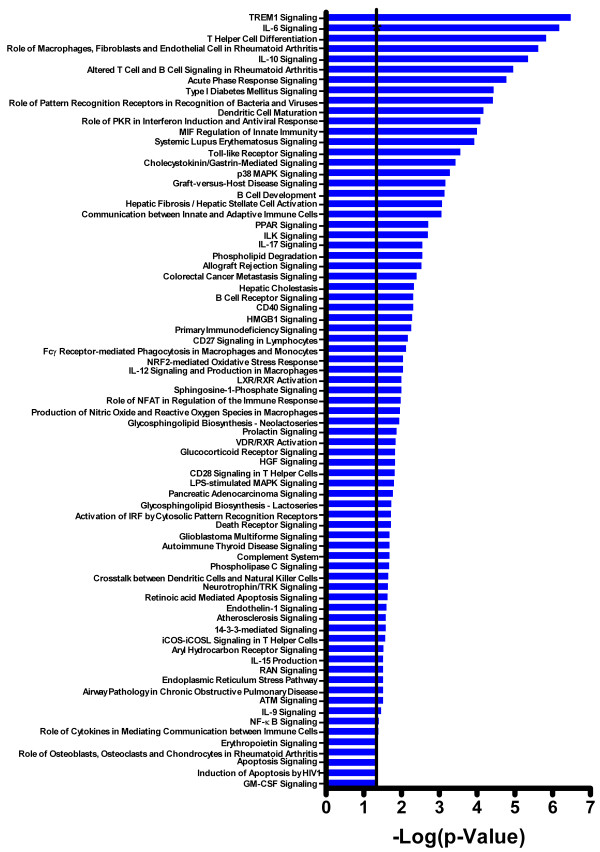
**Canonical pathways significantly altered across all examined brain regions of sarin-exposed seizing animals**. The dataset was filtered on brain region (amygdala, hippocampus, piriform cortex, septum, or thalamus), and a two-way interaction ANOVA was used to identify genes most significantly altered in each brain region based on exposure (saline or sarin) and time after seizure onset. The significant genes from each ANOVA (*p*-value ≤ 0.05) were compared using a Venn diagram, and the top 800 overlapping genes that mapped to canonical pathways in the IPA Knowledge Base were analyzed. The pathways that were significantly affected across all five brain regions are shown (*p *< 0.05, Fisher's exact test), with a threshold of 0.05 (or 1.3 when expressed as -log(p-value)) marked by an asterisk.

**Figure 7:**
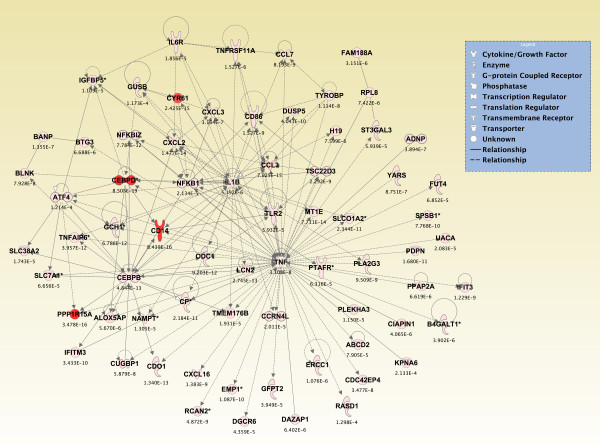
**Seizure-induced alteration of inflammatory response, cellular movement, and hematological system development and function gene network**. The dataset was filtered on brain region (amygdala, hippocampus, piriform cortex, septum, or thalamus), and a two-way interaction ANOVA was used to identify the genes most significantly altered in each region based on exposure (saline or sarin) and time after seizure onset. The significant genes from each ANOVA (*p*-value ≤ 0.05) were compared using a Venn diagram, and the top 800 overlapping genes that mapped to canonical pathways in the IPA Knowledge Base were overlaid onto a global molecular network developed from information within the IPA Knowledge Base. The networks were then algorithmically generated based on their connectivity. Genes are represented as nodes of various shapes to represent the functional class of the gene product, and the biological relationship between two nodes is represented as a line. The intensity of the node color indicates the degree of differential expression.

**Figure 8:**
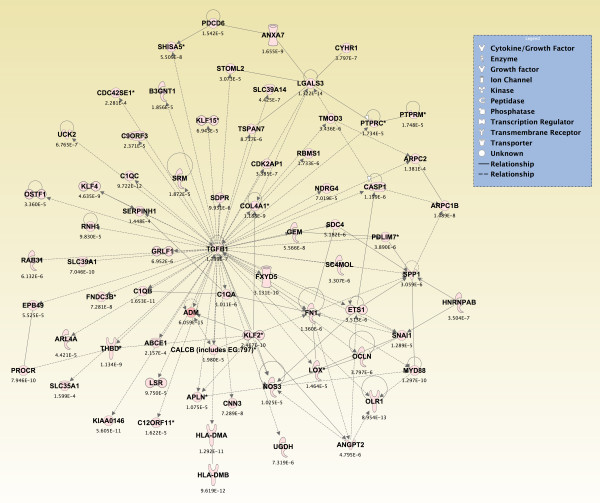
**Seizure-induced alteration of cell-to-cell signalling/interaction, hematological system development/function, and immune cell trafficking gene network**.

**Figure 9:**
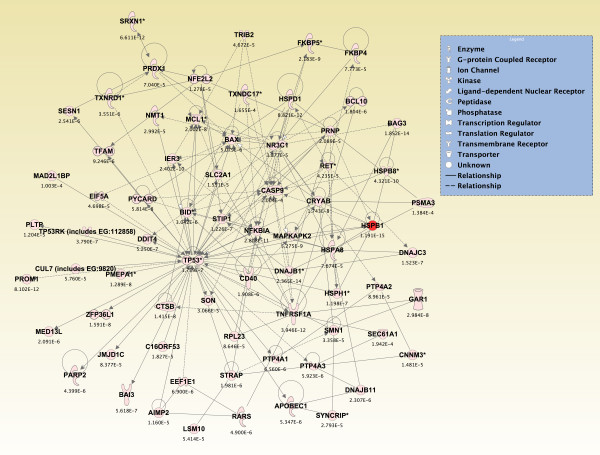
**Seizure-induced alteration of cell morphology, cell death, and organismal survival gene network**.

**Figure 10:**
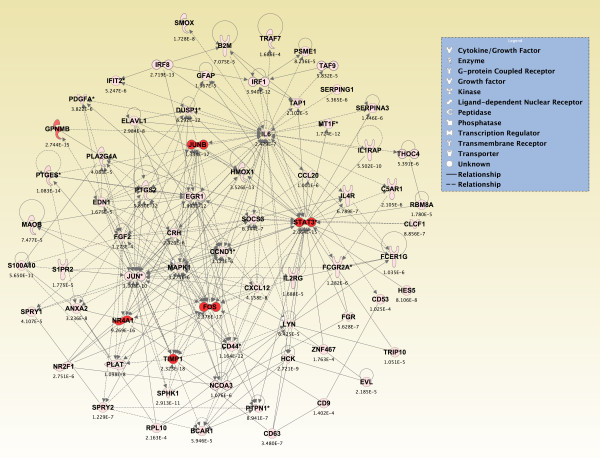
**Seizure-induced alteration of cellular movement, inflammatory response, and cell-to-cell signalling/interaction gene network**.

**Figure 11:**
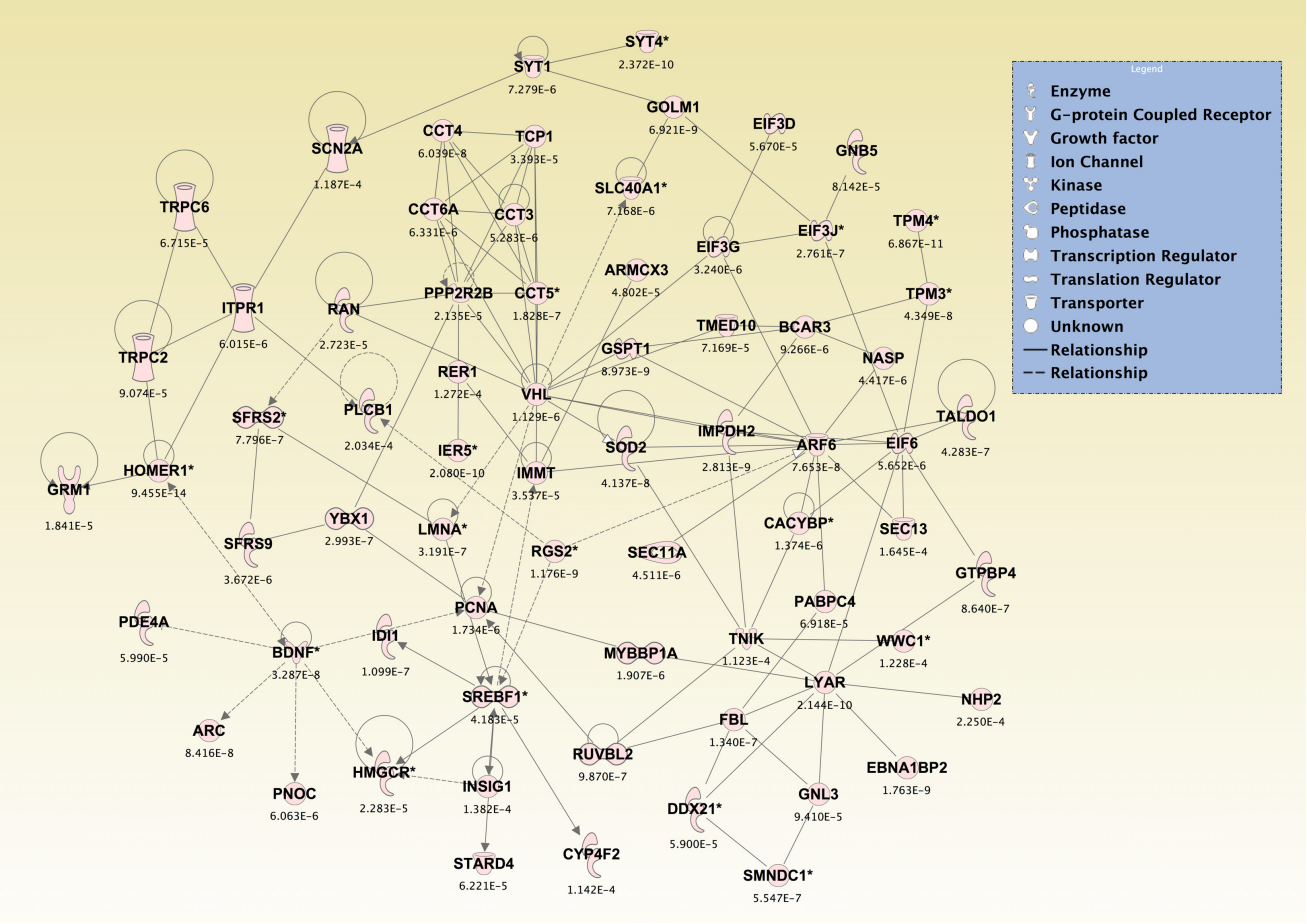
**Seizure-induced alteration of lipid metabolism, small molecule biochemistry, and vitamin/mineral metabolism gene network**.

**Figure 12:**
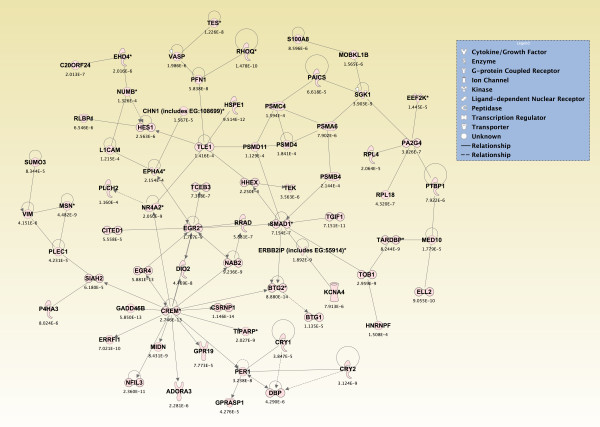
**Seizure-induced alteration of behavior, nervous system development/function, and cellular growth/proliferation gene network**.

Significant genes (*p *≤ 0.05) from the two-way interaction ANOVA for each brain region were compared using a Venn diagram, and the top 800 overlapping genes that mapped to canonical pathways in the IPA Knowledge Base were analyzed (*p *≤ 2.380 × 10^-4^; see Additional File 14). The pathways that were significantly affected across all five brain regions are shown in Figure 6 (*p ≤ *0.05, Fisher's exact test). For *de novo *network generation, these same 800 genes were overlaid onto a global molecular network developed from information within the IPA Knowledge Base. The networks were then algorithmically generated based on their connectivity. The six *de novo *networks identified as being most significantly affected in all five brain regions from sarin-exposed seizing animals are shown in Figures 7, 8, 9, 10, 11 and 12. The first network of genes is built around the pro-inflammatory cytokine TNF-α and is associated with inflammatory response, cellular movement, and hematological system development and function (Figure 7). The second significant network is built around transforming growth factor-beta 1 (TGFβ1) and is associated with cell-to-cell signaling and interaction, hematological system development and function, and immune cell trafficking (Figure 8). The third significant network is built around tumor protein p53 (TP53) and caspase 9 (CASP9). This network is associated with cell morphology, cell death, and organismal survival (Figure 9). The fourth significant network is built around IL-6 and is associated with cellular movement, inflammatory response, and cell-to-cell signaling and interaction (Figure 10). The fifth and sixth significant networks are not built around a specific molecule. The fifth network is associated with lipid metabolism, small molecule biochemistry, and vitamin and mineral metabolism (Figure 11), and the sixth network is associated with behavior, nervous system development and function, and cellular growth and proliferation (Figure 12).

### Multiplexed RT-PCR validation of microarray analysis data

The GeXP genetic analysis system was used to validate a subset of differentially expressed genes in each of the four brain regions. The capillary electrophoresis-based system was used to separate multiplexed RT-PCR reaction products to compare the expression levels of 21 inflammatory cytokines and chemokines in all of the RNA samples. The subset of genes showed relative differences in expression level following sarin-induced seizure that corresponded to expression changes seen in the microarray analysis (see Additional Files 15, 16, 17, 18).

For the amygdala (Additional File 15), the results from both methods showed IL-1α, CCL4, TNF-α, CCL3, and CCL17 expression up-regulated at all five time points (0.25, 1, 3, 6, and 24 h), peaking at 3 h after seizure onset (IL-1α, CCL4, TNF-α, and CCL3) or 6 h after seizure onset (CCL17). CXCL1, CCL7, CCL2, and IL-1ß were also up-regulated at all five time points using both methods. Secretogranin-2 (SCG2), nicotinamide phosphoribosyltransferase (Nampt), and CXCL16 peaked at 6 h after seizure onset before dropping slightly at 24 h in both analyses. Using both technologies, sprouty-related EVH1 domain containing 2 (SPRED2), secreted phosphoprotein-1 (SPP1), and IL-18 expression levels were down-regulated at 0.25 h and 1 h but up-regulated for the remainder of the time course. IL-6 was also slightly down-regulated at 0.25 h and up-regulated for the remainder of the time course with peak expression levels at 3 and 24 h. Using both methods, CXCL10 was down-regulated at 1 and 24 h after seizure onset with peak expression at 6 h post-seizure onset. In both analyses, cardiotrophin-1 (CTF1) expression decreased over the 24-h time course, and CXCL12 was down-regulated throughout the 24-h time course with the largest decrease in expression at 24 h after seizure onset. Ciliary neurotrophic factor (CNTF) and MIF displayed different patterns of expression between the two methods. Using microarray analysis, CNTF expression increased from 0.25 to 3 h, dropped at 6 h, and peaked at 24 h after seizure onset. However, using multiplex RT-PCR, CNTF increased from 0.25 to 6 h and dropped at 24 h. MIF expression decreased throughout the time course using both methods with the exception of a slight increase at 6 h using multiplex RT-PCR.

For the hippocampus (Additional File 16), the results from both methods showed an up-regulation of CCL4 and CCL2 expression at all five time points. TNF-α, CXCL1, CCL3, and IL-1ß were also up-regulated at all time points using both methods; however, the degree of expression increase was not recorded for some time points using multiplex RT-PCR because the transcript levels in the control samples were too low to be detected by the instrument. Using both techniques, CXCL10 was up-regulated at all time points during the time course except for 1 h after seizure onset. CXCL16 was also up-regulated at all time points except 1 h post-seizure onset using both methods. However, the expression increase at 6 h was not determined using multiplex RT-PCR due to control levels not being detected. IL-1α expression was up-regulated at all five time points using multiplex RT-PCR, but appeared to be down-regulated at 0.25 and 24 h using microarray analysis. Using both methods, SCG2 and SPP1 were down-regulated at 0.25 h and up-regulated for the remainder of the time course. CCL7 was also down-regulated at 0.25 h and up-regulated for the remainder of the time course using microarray analysis, but CCL7 expression appeared to be up-regulated at all five time points using multiplex RT-PCR. IL-6 expression was down-regulated at 0.25 and 1 h and increased over the 24-h time period examined using multiplexed RT-PCR. Using microarray analysis, IL-6 expression levels were slightly altered at 0.25 and 1 h, and then up-regulated at 3, 6, and 24 h. SPRED2 expression peaked at 6 h using both methodologies but appeared to be more down-regulated using multiplex RT-PCR. CTF1 expression appeared to be down-regulated at all time points using microarray analysis. It was also down-regulated at all time points except 0.25 h using multiplex RT-PCR. The degree of CTF1 expression decrease was not recorded for 6 and 24 h using multiplex RT-PCR because the transcript levels in the sarin-exposed seizure samples were too low to be detected by the instrument. CNTF, CXCL12, MIF, Nampt, and IL18 expression varied between the two methods. Using microarray analysis, CNTF appeared to be down-regulated at 0.25 h and up-regulated for the remainder of the time course. Using multiplex RT-PCR, CNTF expression appeared to be down-regulated at 0.25, 1, and 6 h, and it appeared to be up-regulated at 3 and 24 h post-seizure onset. CXCL12 and MIF expression decreased over the 24-h time course using microarray analysis. Using multiplex RT-PCR, CXCL12 was down-regulated at 0.25 and 1 h and up-regulated at 3, 6, and 24 h. MIF expression was up-regulated at 0.25, 3, and 6 h and down-regulated at 1 and 24 h. Nampt expression was up-regulated at all five time points except 0.25 h using microarray analysis and 6 h using multiplex RT-PCR. IL-18 expression was down-regulated at the beginning (0.25 and 1 h) of the time course and down-regulated at 3-24 h using microarray analysis. However, IL-18 expression was up-regulated at 0.25 and 1 h and down-regulated at 3-24 h post-seizure onset. CCL17 expression also varied between the two methods. Using microarray analysis, it appeared to be down-regulated at 0.25 and 1 h but up-regulated at 3-24 h. Using multiplex RT-PCR, CCL17 appeared to be up-regulated at all time points except 6 h post-seizure onset. The expression level was not detected in the sarin-exposed seizure hippocampal samples at 6 h, so it appeared to be down-regulated at this time point.

For the septum (Additional File 17), the results from both methods showed an up-regulation of SCG2, SPP1, CCL4, TNF-α, CCL7, CCL2, CCL3, and IL-1ß at all five time points while CXCL12 and CTF1 were down-regulated following sarin-induced seizure, except at the earliest time point of 0.25 h. Using both methods, IL-1α expression peaked at 3 h; SPRED2, IL-18, CXCL10, and CCL17 peaked at 6 h; and CXCL16 peaked at 24 h after seizure onset. CXCL1 expression was up-regulated at all five time points and peaked at 3 h using microarray analysis, but expression changes were not collected using multiplex RT-PCR because transcript levels in the control samples were too low to be detected by the instrument. Using both methods, IL-6 expression appeared to be up-regulated at 3 h, decreased at 6 h, and then peaked at 24 h post-seizure onset. The expression pattern for CNTF, MIF, and Nampt appeared to slightly vary between the two methodologies. CNTF expression appeared to be slightly down-regulated at 0.25 and 6 h using microarray analysis; however, expression was up-regulated using multiplex RT-PCR. MIF expression was down-regulated at 0.25 and 24 h using both methods, but it was also slightly down-regulated at 1 h using microarray analysis and at 3 h using multiplex RT-PCR. Nampt expression appeared to peak at 3 h and slightly decrease at 6 and 24 h using microarray analysis while it appeared to peak at 24 h using multiplex RT-PCR.

For the thalamus (Additional File 18), the results from both methods showed CCL4, TNF-α, CCL7, CCL2, and CXCL10 expression up-regulated and CXCL12 expression down-regulated at all five time points. Nampt and CXCL16 expression levels were also up-regulated at all five time points using multiplexed RT-PCR, but microarray results showed Nampt and CXCL16 down-regulated at 0.25 and 1h, respectively. CXCL1, CCL3, CCL17, and IL-1ß expression levels were up-regulated at all five time points using microarray analysis. Changes in expression were not collected for some time points using multiplex RT-PCR because transcript levels in the control samples were too low to be detected by the instrument, and expression levels were up-regulated at the other time points where data was collected. Therefore, the multiplex RT-PCR data correlates with that from the microarray analysis. IL-1α and IL-6 expression peaked at 3 h after seizure onset using both methods. IL-1α appeared to be down-regulated at 0.25 and 24 h, and IL-6 appeared to be down-regulated at 0.25 and 1 h after seizure onset using multiplex RT-PCR. SPRED2 and SCG2 expression peaked at 6 h, but appeared to be more down-regulated using multiplex RT-PCR. IL-18 expression increased over the 24-h time course with peak expression at 24 h. SPP1 expression levels decreased at 1 h and then continually increased from 3 to 24 h after seizure onset. CNTF and MIF showed slightly different expression patterns using the two different methods. CNTF expression was down-regulated at the first three time points and up-regulated at 6 h using both methods. However, CNTF appeared to be up-regulated at 24 h using microarray analysis and down-regulated at 24 h using multiplex RT-PCR. Using microarray analysis, MIF expression appeared to be up-regulated at 0.25 and 3 h, down-regulated at 1 and 6 h, and unchanged at 24 h. Using multiplex RT-PCR, MIF expression was up-regulated at 0.25 and 1 h, down-regulated at 3 and 6 h, and then up-regulated again at 24 h post-seizure onset. CTF1 expression also appeared to differ between the two methods. Using microarray analysis, CTF1 appeared to be down-regulated at the beginning (0.25 h) and end (24 h) of the time course and up-regulated at 1, 3, and 6 h after seizure onset. Using multiplex RT-PCR, expression data was not collected for CTF1 at 0.25 h because transcript levels in the control samples were too low to be detected by the GeXP instrument but was up-regulated at 1, 3, and 24 h and down-regulated at 6 h after seizure onset.

Although there are some variations in expression data between the two methods, the overall expression levels of the examined inflammatory cytokines and chemokines were in close agreement using the two different methodologies. The differences in expression levels can easily arise from variation in quantitative methodology. Because these two technologies are different, slight differences in expression between the two methods are expected, but overall the data between the microarray analysis and multiplexed PCR analysis are in close agreement.

## Discussion

In this study, we performed gene expression profiling to assess the temporal transcriptional changes associated with sarin-induced seizure. Comparison of the transcriptional profiles of five nerve agent-sensitive rat brain regions (amygdala, hippocampus, piriform cortex, septum, and thalamus) indicated the presence of a robust inflammatory response in sarin-exposed seizing animals. In agreement with previously reported findings [13,18-26], we observed a rapid activation of the innate immune response that persisted throughout the 24-h time period examined. Therefore, we predict that pro-inflammatory cytokines and their signaling pathways could potentially mediate some of the molecular and structural changes observed after nerve agent-induced seizure activity. Because current countermeasures do not fully prevent neuropathology, particularly in scenarios where treatment is delayed (e.g., civilian terrorist attack), identifying therapeutic targets that mediate the cascade of secondary events leading to brain damage and functional impairment is critical.

Our findings, which are in agreement with previous studies of soman-induced brain damage [1,11,18,20,27], indicate that sarin toxicity correlates with the development and duration of seizures. Significant changes in gene expression were seen in all five brain regions following seizure occurrence with the greatest effects in the piriform cortex, which is in agreement with the findings of Lemercier at al. [28] and McDonough et al. [10]. However, it should be noted that a higher vulnerability to nerve agent-induced brain damage has been observed in other cerebral regions of other animal models. These highly susceptible brain regions include the thalamus in mice [11], the amygdala in guinea pigs [27], and the frontoparietal cortex and cerebellum in monkeys [29,30]. This variability may be due to interspecies differences in functional anatomy and/or differences in study design. However, the five brain regions examined in our study are nerve agent sensitive regions of the rat brain. Therefore, we examined each of these brain regions for common nerve agent-induced pathways to explore molecular mechanisms involved in nerve agent-induced brain injury.

Gene ontology analysis revealed numerous canonical pathways that were significantly altered by sarin-induced seizure in the five regions of the rat brain examined in this study. The pathways that were significantly affected in all five brain regions are known to be associated with an inflammatory response and include many pro-inflammatory cytokines. These pathways include ATM signaling, CD40 signaling, IL-10 signaling, IL-6 signaling, MIF regulation of innate immunity, role of double-stranded PKR in interferon induction and antiviral response, toll-like receptor signaling, and TREM1 signaling. Furthermore, pro-inflammatory cytokines were among the *de novo *networks identified as most significantly affected in all five brain regions from sarin-exposed seizing animals. Two of the top six networks were associated with an inflammatory response. One network of genes was built around TNF-α as a central node and the other around IL-6 as a central node.

Pro-inflammatory cytokines are known to mediate cellular communication and play a significant role in the pathological processes involved in various brain diseases, such as SE [31-35]. Although picomolar or low nanomolar ranges of cytokines enhance neuronal survival, higher concentrations have deleterious effects on neuronal viability [36]. The findings presented in this study are in agreement with the observed inflammatory response previously reported following nerve agent intoxication [13,18-26], as well as in other models of seizure induction [33,37]. Although these studies all show an up-regulation of inflammatory cytokines, it should be noted that the expression patterns of individual cytokines vary from study to study. These variations likely stem from differences in the nerve agent tested (sarin vs soman), animal models used (rats vs mice), pharmacological treatments given, and, maybe most importantly, seizure intensity [24]. For example, Williams et al. [19] and Dhote et al. [24] employed quantitative real time-PCR to characterize the temporal response of inflammatory cytokines following soman-induced seizures in rats and mice, respectively. Williams and colleagues showed an initial up-regulation of TNF-α mRNA in the hippocampus, piriform cortex, and thalamus at their earliest time point of 2 h after exposure. This was subsequently followed by an increase in IL-1β and IL-6 mRNA at 6 h after exposure. The findings of Dhote et al. [24] also indicated a significant induction of inflammatory cytokines in the whole cortex and hippocampus but not in the cerebellum. Cytokine activation was seen as early as 1 h after exposure in the cortex with a peak response between 6 and 24 h. However, cytokine up-regulation was delayed to 6 h after exposure in the hippocampus with peak expression levels observed between 24 and 48 h. Our data also show an acute up-regulation of inflammatory cytokines, but the temporal expression patterns are slightly different. We observed a significant increase in IL-1β, IL-6, and TNF-α expression levels as early as 0.25 h following seizure onset in all five brain regions examined. Of the three cytokines listed, IL-1β expression peaked the earliest following seizure onset and dropped to near control levels in the hippocampus, septum, and thalamus by 24 h, whereas it appeared to be leveling out in the amygdala and increasing in the piriform cortex at 24 h. It is known that IL-1β induces its own synthesis [38], and this positive feedback loop could enhance and extend the IL-1β response [33]. Therefore, we hypothesize that this positive feedback loop could be a contributing factor in the greater neuropathology seen in the piriform cortex of the rat model. The peak in IL-1β expression is followed by IL-6 and TNF-α, which both peaked at 3 h in all five brain regions. Moynagh et al. [39] showed that IL-1β stimulates the expression of NF-κB, which in turn activates a variety of genes involved in the inflammatory response in CNS diseases [33]. Therefore, our data appear to support the role of NF-κB in the neuropathology resulting from nerve agent-induced seizures as it shows a sharp induction by 3 h after seizure onset in all brain regions examined (data not shown) along with IL-6 and TNF-α expression. TNF-α expression decreased to near control levels by 24 h in all five brain regions. However, IL-6 expression decreased at 6 h in all five brain regions and then increased at 24 h after seizure onset in all regions except the thalamus.

Studies have shown that nerve agent-induced seizures initially result from AChE inhibition and overstimulation of cholinergic receptors, which is followed by an increase of excitatory amino acids (EAAs), such as glutamate [1-3]. This glutamatergic hyperactivity causes an opening of N-methyl-D-aspartate (NMDA) calcium channels and a subsequent increase in intracellular calcium [1,40,41], which in turn initiates signaling cascades that cause neuronal death. Based on our findings and those of other investigators, it appears that inflammatory signaling pathways are an important component of nerve agent-induced brain injury; however, the molecular mechanisms by which nerve agent-induced seizures produce acute neuroinflammation or how this phenomenon contributes to the ensuing neuropathology following exposure is still unclear. *In vitro *studies have shown that pro-inflammatory cytokines play a role in glutamate toxicity as they inhibit glial cells from taking up excess extracellular glutamate [42]. Cytokines are thought to further enhance this glutamatergic hyperactivity by increasing NMDA receptor activity [43], which promotes excitotoxic neuronal cell death [44,45]. Another mechanism by which pro-inflammatory cytokines could contribute to nerve agent toxicity involves blood-brain barrier (BBB) function. The brain is normally isolated from the peripheral immune system via the BBB; however, a neurotoxic insult, such as a convulsant dose of sarin, can induce both a local and peripherally recruited inflammatory response. BBB damage has been seen in many neurodegenerative diseases and animal models of seizure [46], and studies have shown that pro-inflammatory cytokines including TNF-α, IL-1β, IL-6, and interferon-λ are implicated in the regulation of BBB permeability [47-49]. For example, IL-1β can affect the permeability of the BBB via disruption of the tight-junction organization or production of nitric oxide and matrix metalloproteinases in endothelial cells [49]. The data from our sarin exposure model indicate that there could be a breakdown of the BBB as well. This was indicated by the up-regulation of inter-cellular adhesion molecule-1 (ICAM1) and E-selectin in all five brain regions. These molecules are thought to be linked to signal transduction cascades leading to junctional reorganization as they can interact with the actin cytoskeleton, which in turn is an indicator of infiltration of peripheral leukocytes into damaged brain regions through the BBB [47]. We also observed a decrease in occludin expression levels, which is one of the main components of tight junctions, indicating a possible loss of tight junction integrity. The findings of our study support those of Abdel-Rahman et al. [50] who demonstrated that a toxic dose of sarin can break down the BBB and speculated that this disruption plays an important role in sarin-induced cell death in the motor cortex, hippocampus, and cerebellum. Furthermore, Damodaran et al. [22,23] identified numerous BBB-related genes that were altered at 15 min and persisted until three months following 0.5 and 1.0 × LD_50 _sarin exposure.

## Conclusions

Transcriptional profiling and pathway modeling provide a powerful approach in the development of neuroprotectants against nerve agent exposure. The findings of this study are in agreement with those previously reported and provide critical insight into the identification of possible therapeutic targets that could potentially provide broad-spectrum protection in areas of the central nervous system known to be damaged by nerve agent-induced seizure. The persistently altered expression profiles of pro-inflammatory cytokines strongly suggests that they may play a role in the sequence of molecular events leading to nerve agent-induced neuropathological changes. Therefore, the antagonism of pro-inflammatory molecules, as well as their receptors and signaling pathways, may represent novel targets for the development of drug therapies that would alleviate neurological damage when given after the onset of seizures and secondary responses that lead to brain injury have begun.

## List of abbreviations

OP: organophosphorus; AChE: acetylcholinesterase; ACh: acetylcholine; SE: status epilepticus; USAMRICD: United States Army Medical Research Institute of Chemical Defense; EEG: electroencephalogram; 2-PAM: 2-pyridine aldoxime methylchloride; MIAME: Minimal Information About a Microarray Experiment; GEO: Gene Expression Omnibus; RMA: robust multiarray averaging; PCA: principal component analysis; ANOVA: analysis of variance; IPA: Ingenuity Pathways Analysis; GeXP: GenomeLab Gene Expression Profiler; GAPDH: glyceraldehyde 3-phosphate dehydrogenase; Kan^r^: kanamycin resistance; RFU: relative fluorescence units; A.U.: arbitrary units; ATM: ataxia telangiectasia mutated protein; IL: interleukin; MIF: macrophage migration inhibitory factor; PKR: RNA-activated protein kinase; TREM1: triggering receptor expressed on myeloid cells 1; TNF-α: tumor necrosis factor-α; THOP1: thimet oligopeptidase 1; ERK: extracellular receptor kinase; MAPK: mitogen activated protein kinase; FGF: fibroblast growth factor; PPARα: peroxisome proliferator-activated receptor-α; RXRα: retinoid × receptor-α; IRF: interferon-regulatory factor; GM-CSF: granulocyte-macrophage colony-stimulating factor; NF-κB: nuclear factor kappa-light-chain-enhancer of activated B cells; PDGF: platelet-derived growth factor; NFAT: role of nuclear factor of activated T cells; VDR: vitamin D receptor; RXR: retinoic acid × receptor; TGF-β: transforming growth factor-β; TP53: tumor protein p53; CASP: caspase; CCL: chemokine (C-C motif) ligand; CXCL: chemokine (C-X-C) motif ligand; SCG2: secretogranin-2; Nampt: nicotinamide phosphoribosyltransferase; SPRED2: sprouty-related, EVH1 domain containing 2; SPP1: secreted phosphoprotein-1; CTF1: cardiotrophin-1; CNTF: ciliary neurotrophic factor; CNS: central nervous system; EEA: excitatory amino acid; NMDA: N-methyl-D-aspartate; BBB: blood-brain barrier; ICAM-1: inter-cellular adhesion molecule-1

## Competing interests

The authors declare that they have no competing interests.

## Authors' contributions

KDS collected and processed brain regions for microarray and GeXP analysis, analyzed all data, and drafted the manuscript. LAL participated in developing and coordinating the study. CLR conducted behavioral assessments of the animals. JLM dissected brain regions from all animals. JFD participated in the brain dissections, study design, data analysis, and helped draft the manuscript. All authors read and approved the final manuscript.

## Supplementary Material

Additional file 1 **Gene symbol, PCR product size, and primer sequences used in multiplex RT-PCR assays**. Click here for file

Additional file 2 **Concentrations of reverse primers within the RT-PCR multiplex**. Click here for file

Additional file 3 **Top 800 significantly altered genes in piriform cortex post-seizure onset used for gene ontology analysis**. Click here for file

Additional file 4 **Top 800 significantly altered genes in hippocampus post-seizure onset used for gene ontology analysis**. Click here for file

Additional file 5 **Top 800 significantly altered genes in amygdala post-seizure onset used for gene ontology analysis**. Click here for file

Additional file 6 **Top 800 significantly altered genes in septum post-seizure onset used for gene ontology analysis**. Click here for file

Additional file 7 **Top 800 significantly altered genes in thalamus post-seizure onset used for gene ontology analysis**. Click here for file

Additional file 8 **Canonical pathways significantly altered in sarin-exposed seizing animals**. A two-way interaction ANOVA was performed to identify genes most significantly altered in each brain region based on exposure (saline or sarin) and time after seizure onset. The top 800 genes ranked by *p*-value were mapped to canonical pathways in the IPA Knowledge Base to identify molecular effects in seizing animals. Only those canonical pathways that were significantly affected in at least one brain region are shown (*p *< 0.05, Fisher's exact test); the -log of the *p*-value is shown for each pathway (1.3 = -log of 0.05). Pink indicates detected and significant (≥ 1.3); grey indicates detected but not significant (< 1.3); white indicates not detected. Pathways significant for all brain regions are indicated in red. Click here for file

Additional file 9 **Canonical pathways significantly altered only in the amygdala of sarin-exposed seizing animals**. A two-way interaction ANOVA was performed to identify genes significantly altered based on exposure (saline or sarin) and time after seizure onset. The top 800 genes (ranked by *p*-value) that mapped to canonical pathways in the IPA Knowledge Base were used to identify molecular effects in the amygdala of seizing animals. The canonical pathways that were significantly affected only in the amygdala (and not in any of the other brain regions examined) are shown. To further characterize these pathways, we filtered the data again based on time, and an ANOVA was performed to identify genes significantly altered based on exposure (saline or sarin) at each time point following seizure onset. When the data was analyzed at individual time points, we found that apoptosis signaling was significant at 6 h; induction of apoptosis by HIV1 was significant at 3 and 6 h; karatan sulfate biosynthesis was significant at 0.25, 3, and 6 h; and role of osteoblasts, osteoclasts, and chondrocytes in rheumatoid arthritis was not significant at any of the time points analyzed. The -log of the *p*-value are graphed for each time point (1.3 = -log of 0.05). Click here for file

Additional file 10 **Canonical pathways significantly altered only in the hippocampus of sarin-exposed seizing animals**. When the data was analyzed at individual time points, we found that acute myeloid leukemia signaling and thyroid cancer signaling were significant at 3 h; urea cycle and metabolism of amino groups was significant at 24 h; and hypoxia signaling in the cardiovascular system was significant at 1, 3, and 24 h following seizure onset. Neuroprotective role of THOP1 in Alzheimer's disease and retinoic acid mediated apoptosis signaling were not significant at any of the individual time points examined. Click here for file

Additional file 11 **Canonical pathways significantly altered only in the piriform cortex of sarin-exposed seizing animals**. When analyzed at individual time points, we found that G-protein coupled receptor signaling and PPARα/RXRα activation were significant at 1 h; glioma invasiveness signaling and taurine and hypotaurine metabolism were significant at 24 h; ERK/MAPK signaling was significant at 1 and 24 h; and IL-1 signaling was significant at 3 and 6 h. FGF signaling, glycerophospholipid metabolism, and neuropathic pain signaling in dorsal horn neurons were not significant at any time point analyzed. Click here for file

Additional file 12 **Canonical pathway significantly altered only in the septum of sarin-exposed seizing animals**. When analyzed at individual time points, it was significant only at 24 h after seizure onset. Click here for file

Additional file 13 **Canonical pathways significantly altered only in the thalamus of sarin-exposed seizing animals**. When the data was analyzed at individual time points, we found that primary immunodeficiency signaling was significant at 6 h; autoimmune thyroid disease, glioblastoma multiforme signaling, iCOS-iCOSL signaling in T helper cells, and phospholipase C signaling were significant at 24 h; estrogen-dependent breast cancer signaling was significant at 0.25 and 3 h; aryl hydrocarbon receptor signaling was significant at 3 and 6 h; B cell receptor signaling was significant at 0.25, 6, and 24 h; PDGF signaling was significant at 1, 3, and 6 h; VDR/RXR activation was significant at 1, 6, and 24 h; crosstalk between dendritic cells and natural killer cells was significant at 3, 6, and 24 h; p53 signaling was significant at 0.25, 3, 6, and 24 h; and molecular mechanisms of cancer was significant at 1, 3, 6, and 24 h after seizure onset. The remaining pathways were not significantly altered in the thalamus at any individual time point following sarin-induced seizure onset. Click here for file

Additional file 14 **Top 800 significantly altered genes across all brain regions from sarin-exposed seizing animals**. Click here for file

Additional file 15 **Microarray analysis and multiplexed RT-PCR show similar gene expression changes in amygdala following sarin-induced seizure**. The GeXP genetic analysis system was used to measure the expression levels of 21 differentially expressed cytokines or chemokines by multiplexed RT-PCR to validate the microarray data. The expression of each gene within a sample was normalized to GAPDH expression to minimize inter-capillary variation, and the normalized intensity of each replicate (n ≥ 3) was used to calculate an average intensity of each sample group (i.e. control or sarin-induced seizure at each time point). The fold expression difference between control and sarin-induced seizure samples is shown for each gene at each of the five time points examined. The fold changes in expression obtained in the microarray analysis are shown on the left, and the fold changes in expression obtained in the multiplex PCR analysis are shown on the right. Genes that were down-regulated following sarin-induced seizure are shaded in green, and genes that were up-regulated following sarin-induced seizure are shaded in red. Changes in expression were not collected for some time points using multiplex RT-PCR because transcript levels in the control samples were too low to be detected by the instrument (indicated by an asterisk). Click here for file

Additional file 16 **Microarray analysis and multiplexed RT-PCR show similar gene expression changes in hippocampus following sarin-induced seizure**. Click here for file

Additional file 17 **Microarray analysis and multiplexed RT-PCR show similar gene expression changes in septum following sarin-induced seizure**. Click here for file

Additional file 18 **Microarray analysis and multiplexed RT-PCR show similar gene expression changes in thalamus following sarin-induced seizure**. Click here for file

## References

[B1] McDonough JH Jr Shih TM Neuropharmacological mechanisms of nerve agent-induced seizure and neuropathology Neurosci Biobehav Rev 1997 21 559 579 10.1016/S0149-7634(96)00050-4 9353792

[B2] Bajgar J Complex view on poisoning with nerve agents and organophosphates Acta Medica (Hradec Králové) 2005 48 1 3 21 16080378

[B3] Aroniadou-Anderjaska V Figueiredo TH Apland JP Qashu F Braga MFM Primary brain targets of nerve agents: the role of the amygdala in comparison to the hippocampus Neurotoxicology 2009 30 772 776 10.1016/j.neuro.2009.06.011 19591865 PMC2761531

[B4] Shih TM Duniho SM McDonough JH Jr Control of nerve agent-induced seizures is critical for neuroprotection and survival Toxicol Appl Pharmacol 2003 188 69 80 10.1016/S0041-008X(03)00019-X 12691725

[B5] Myhrer T Enger S Aas P Efficacy of immediate and subsequent therapies against soman-induced seizures and lethality in rats Basic Clin Pharmacol Toxicol 2006 98 184 191 10.1111/j.1742-7843.2006.pto_268.x 16445593

[B6] Myhrer T Neuronal structures involved in the induction and propagation of seizures caused by nerve agents: implications for medical treatment Toxicology 2007 239 1-2 1 14 10.1016/j.tox.2007.06.099 17689166

[B7] Brown MA Brix KA Review of health consequences from high-, intermediate and low-level exposure to organophosphorus nerve agents J Appl Toxicol 1998 18 393 408 10.1002/(SICI)1099-1263(199811/12)18:6<393::AID-JAT528>3.0.CO;2-0 9840747

[B8] Bajgar J Ševelová L Krejčová G Fusek J Vachek J Kassa J Herink J Biochemical and behavioral effects of soman vapors in low concentrations Inhal Toxicol 2004 16 497 507 10.1080/08958370490442430 15204741

[B9] Myhrer T Enger S Aas P Anticonvulsant effects of damage to structures involved in seizure induction in rats exposed to soman Neurotoxicology 2007 28 819 828 10.1016/j.neuro.2007.03.010 17512981

[B10] McDonough JH Jr Dochterman LW Smith CD Shih TM Protection against nerve agent induced neuropathology, but not cardiac pathology, is associated with the anti-convulsant action of drug treatment Neurotoxicology 1995 15 123 132 7603632

[B11] Baille V Clarke PGH Brochier G Dorandeu F Verna JM Four E Lallement G Carpentier P Soman-induced convulsions: the neuropathology revisited Toxicology 2005 215 1 24 10.1016/j.tox.2005.05.028 16054742

[B12] Angoa-Pérez M Kreipke CW Thomas DM Van Shura KE Lyman M McDonough JH Kuhn DM Soman increases neuronal COX-2 levels: possible link between seizures and protracted neuronal damage Neurotoxicology 2010 31 738 746 10.1016/j.neuro.2010.06.007 20600289 PMC2974036

[B13] Zimmer LA Ennis M Shipley MT Soman-induced seizures rapidly activate astrocytes and microglia in discrete brain regions J Comp Neurol 1997 378 482 492 10.1002/(SICI)1096-9861(19970224)378:4<482::AID-CNE4>3.0.CO;2-Z 9034905

[B14] Racine RJ Modification of seizure activity by electrical stimulation. II. Motor seizure Electroencephalogr Clin Neurophysiol 1972 32 3 281 294 10.1016/0013-4694(72)90177-0 4110397

[B15] Dillman JF III Phillips CS Dorsch LM Croxton MD Hege AI Sylvester AJ Moran TS Sciuto AM Genomic analysis of rodent pulmonary tissue following bis-(2-chloroethyl) sulfide exposure Chem Res Toxicol 2005 18 28 34 10.1021/tx049745z 15651846

[B16] Irizarry RA Bolstad BM Collin F Cope LM Hobbs B Speed TP Summaries of Affymetrix GeneChip probe level data Nucleic Acids Res 2003 31 4 1 8 12582260 10.1093/nar/gng015 PMC150247

[B17] Pearson K On lines and planes of closest fit to systems of points in space Philos Mag 1901 2 559 572

[B18] Svensson I Waara L Johansson L Bucht A Cassel G Soman-induced interleukin-1β mRNA and protein in rat brain Neurotoxicology 2001 22 355 362 10.1016/S0161-813X(01)00022-5 11456336

[B19] Williams AJ Berti R Yao C Price RA Velarde LC Koplovitz I Schultz SM Tortella FC Dave JR Central neuro-inflammatory gene response following soman exposure in the rat Neurosci Lett 2003 349 147 150 10.1016/S0304-3940(03)00818-8 12951190

[B20] Svensson I Waara L Cassel G Effects of HI 6, diazepam and atropine on soman-induced IL-1β protein in rat brain Neurotoxicology 2005 26 173 181 10.1016/j.neuro.2004.11.004 15713338

[B21] Chapman S Kadar T Gilat E Seizure duration following sarin exposure affects neuro-inflammatory markers in the rat brain Neurotoxicology 2006 27 277 283 10.1016/j.neuro.2005.11.009 16406030

[B22] Damodaran TV Greenfield ST Patel AG Dressman HK Lin SK Abou-Donia MB Toxicogenomic studies of the rat brain at an early time point following acute sarin exposure Neurochem Res 2006 31 367 381 10.1007/s11064-005-9023-5 16733813

[B23] Damodaran TV Patel AG Greenfield ST Dressman HK Lin SM Abou-Donia MB Gene expression profiles of the rat brain both immediately and 3 months following acute sarin exposure Biochem Pharmacol 2006 71 497 520 10.1016/j.bcp.2005.10.051 16376859

[B24] Dhote F Peinnequin A Carpentier P Baille V Delacour C Foquin A Lallement G Dorandeu F Prolonged inflammatory gene response following soman-induced seizures in mice Toxicology 2007 238 166 176 10.1016/j.tox.2007.05.032 17662515

[B25] Dillman JF III Phillips CS Kniffin DM Tompkins CP Hamilton TA Kan RK Gene expression profiling of rat hippocampus following exposure to the acetylcholinesterase inhibitor soman Chem Res Toxicol 2009 22 4 633 638 10.1021/tx800466v 19281266

[B26] Johnson EA Kan RK The acute phase response and soman-induced status epilepticus: temporal and regional changes in rat brain cytokine concentrations J Neuroinflammation 2010 7 40 1 9 20649973 10.1186/1742-2094-7-40 PMC2914669

[B27] Carpentier P Foquin A Rondouin G LernerNatoli M deGroot DMG Lallement G Effects of atropine sulphate on seizure activity and brain damage produced by soman in guinea pigs: ECoG correlates of neuropathology Neurotoxicology 2000 4 521 540 11022861

[B28] Lemercier G Carpentier P Sentenac-Roumanou H Moralis P Histological and histochemical changes in the central nervous system of the rat poisoned by an irreversible anticholinesterase organophosphorus compound Acta Neuropathol (Berlin) 1983 61 123 129 10.1007/BF00697391 6637396

[B29] Lallement G Mestries JC Privat A Brochier G Baubichon D Carpentier P Kamenka JM Sentenac-Roumanou H Burckhart MF Peoc'h M GK 11: promising additional neuroprotective therapy for organophosphate poisoning Neurotoxicology 1997 18 3 851 856 9339831

[B30] Lallement G Clarencon D Masqueliez C Baubichon D Galonnier M Burckhart MF Peoc'h M Mestries JC Nerve agent poisoning in primates: antilethal, anti-epileptic and neuroprotective effects of GK-11 Arch Toxicol 1998 72 2 84 92 9456079 10.1007/s002040050472

[B31] De Simoni MG Perego C Ravizza T Moneta D Conti M Marchesi F De Luigi A Garattini S Vezzani A Inflammatory cytokines and related genes are induced in the rat hippocampus by limbic status epilepticus Eur J Neurosci 2000 12 2623 2633 10.1046/j.1460-9568.2000.00140.x 10947836

[B32] Viviani B Bartesaghi S Corsini E Galli CL Marinovich M Cytokines role in neurodegenerative events Toxicol Lett 2004 149 85 89 10.1016/j.toxlet.2003.12.022 15093252

[B33] Voutsinos-Porche B Koning E Kaplan H Ferrandon A Guenounou M Nehlig A Motte J Temporal patterns of the cerebral inflammatory response in the rat lithium-pilocarpine model of temporal lobe epilepsy Neurobiol Dis 2004 17 385 402 10.1016/j.nbd.2004.07.023 15571975

[B34] Vezzani A Inflammation and epilepsy Curr Rev Basic Sci 2005 5 1 1 6 10.1111/j.1535-7597.2005.05101.x PMC1176317 16059445

[B35] Choi J Koh S Yonsei Med J 2008 49 1 1 18 10.3349/ymj.2008.49.1.1 18306464 PMC2615265

[B36] Morganti-Kossmann MC Kossmann T Wahl SM Cytokines and neuropathology Trends Pharmacol Sci 1992 13 286 291 1509523 10.1016/0165-6147(92)90087-m

[B37] Lehtimaki KA Peltola J Koskikallio E Keranen T Honkaniemi J Expression of cytokines and cytokine receptors in the rat brain after kainic acid-induced seizures Mol Brain Res 2003 110 253 260 10.1016/S0169-328X(02)00654-X 12591161

[B38] Dinarello CA Ikejima T Warner SJ Orencole SF Lonnemann G Cannon JG Libby P Interleukin 1 induces interleukin 1. I. Induction of circulating interleukin 1 in rabbits *in vivo *and in human mononuclear cells *in vitro* J Immunol 1987 139 1902 1910 3497982

[B39] Moynagh PN Williams DC O'Neill LA Interleukin-1 activates transcription factor NFκB in glial cells Biochem J 1993 294 343 347 8373349 10.1042/bj2940343 PMC1134460

[B40] Berridge MJ Unlocking the secrets of cell signaling Annu Rev Physiol 2005 67 1 21 10.1146/annurev.physiol.67.040103.152647 15709950

[B41] Filbert M Levine E Ballough G Neuroprotection for nerve agent-induced brain damage by blocking delayed calcium overload: a review J Med Chem Biol Radiol Def 2005 3 1 21

[B42] Ye ZC Sontheimer H Cytokine modulation of glial glutamate uptake: A possible involvement of nitric oxide Neuroreport 1996 7 13 2181 2185 10.1097/00001756-199609020-00025 8930985

[B43] Viviani B Bartesaghi S Gardoni F Vezzani A Behrens MM Bartfai T Binaglia M Corsini E Di Luca M Galli CL Marinovich M Interleukin-1β enhances NMDA receptor-mediated intracellular calcium increase through activation of the Src family of kinases J Neurosci 2003 23 8692 8700 14507968 10.1523/JNEUROSCI.23-25-08692.2003 PMC6740426

[B44] Ravizza T Lucas SM Balosso S Bernardino L Ku G Noé F Malva J Randle JCR Allan S Vezzani A Inactivation of caspase-1 in rodent brain: a novel anticonvulsive strategy Epilepsia 2006 47 7 1160 1168 10.1111/j.1528-1167.2006.00590.x 16886979

[B45] Ravizza T Gagliardi B Noé F Boer K Aronica E Vezzani A Innate and adaptive immunity during epileptogenesis and spontaneous seizures: evidence from experimental models and human temporal lobe epilepsy Neurobiol Dis 2008 29 142 160 10.1016/j.nbd.2007.08.012 17931873

[B46] Carvey PM Hendey B Monahan AJ The blood-brain barrier in neurodegenerative disease: a rhetorical perspective J Neurochem 2009 111 2 291 314 10.1111/j.1471-4159.2009.06319.x 19659460 PMC2761151

[B47] Bolton SJ Anthony DC Perry VH Loss of the tight junction proteins occludin and zonula occludens-1 from cerebral vascular endothelium during neutrophil-induced blood-brain barrier breakdown *in vivo* Neurosci 1998 86 4 1245 1257 10.1016/S0306-4522(98)00058-X 9697130

[B48] Mayhan WG Cellular mechanisms by which tumor necrosis factor-α produces disruption of the blood-brain barrier Brain Res 2002 927 144 152 10.1016/S0006-8993(01)03348-0 11821008

[B49] Allan SM Tyrrell PJ Rothwell NJ Interleukin-1 and neuronal injury Nat Rev Immunol 2005 5 8 629 640 10.1038/nri1664 16034365

[B50] Abdel-Rahman A Shetty AK Abou-Donia MB Acute exposure to sarin increases blood brain barrier permeability and induces neuropathological changes in the rat brain: dose-response relationships Neurosci 2002 113 3 721 741 10.1016/S0306-4522(02)00176-8 12150792

[B51] Spradling in press 21777429

