# Supplementary figures and images for: Transcriptional responses of the nerve agent-sensitive brain regions amygdala, hippocampus, piriform cortex, septum, and thalamus following exposure to the organophosphonate anticholinesterase sarin
Source: J Neuroinflammation. 2011 Jul 21;8:84. doi: 10.1186/1742-2094-8-84 (PMC3180277; doi:10.1186/1742-2094-8-84)

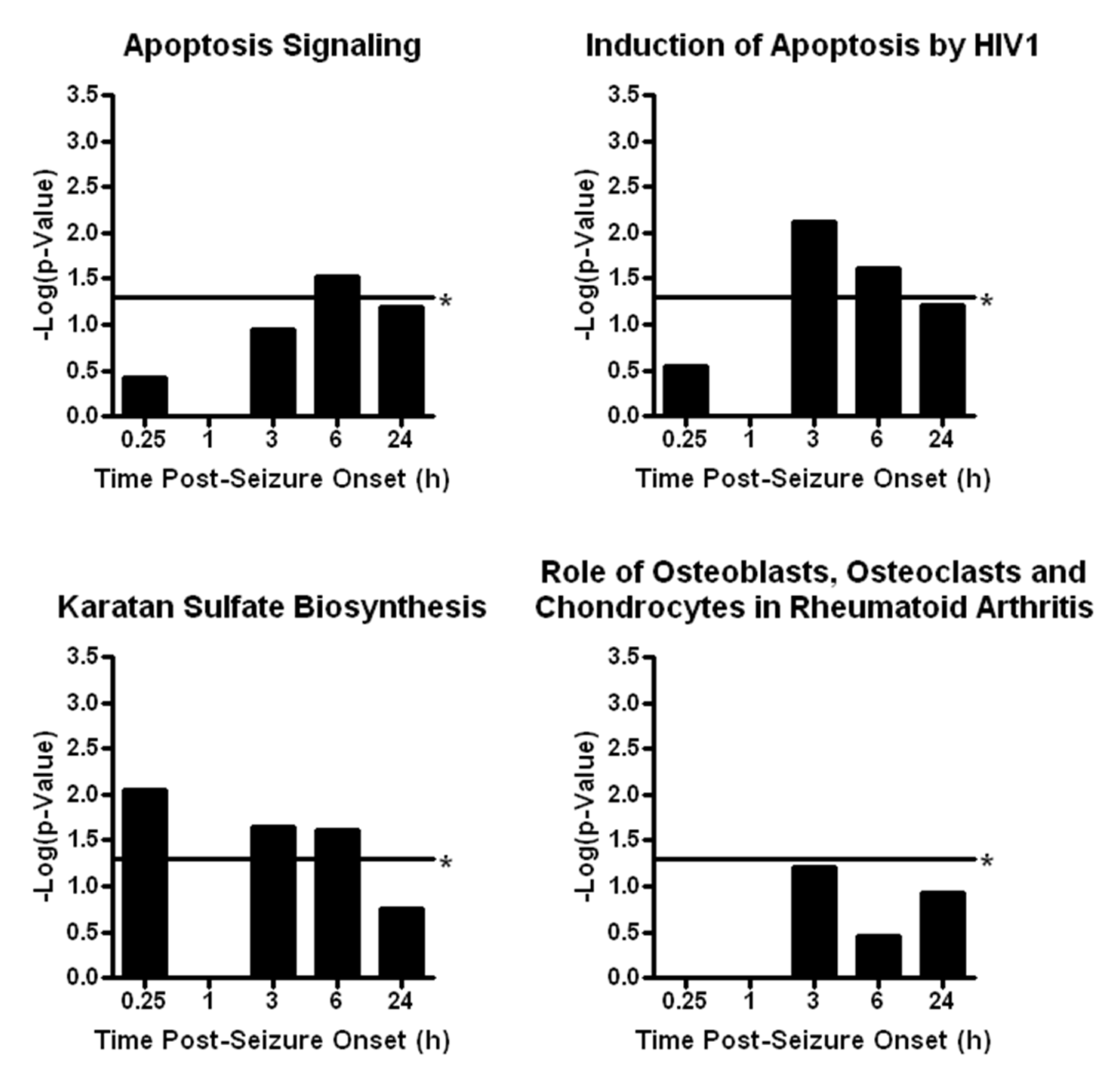

Supplement: Additional file 9 — Canonical pathways significantly altered only in the amygdala of sarin-exposed seizing animals. A two-way interaction ANOVA was performed to identify genes significantly altered based on exposure (saline or sarin) and time after seizure onset. The top 800 genes (ranked by p-value) that mapped to canonical pathways in the IPA Knowledge Base were used to identify molecular effects in the amygdala of seizing animals. The canonical pathways that were significantly affected only in the amygdala (and not in any of the other brain regions examined) are shown. To further characterize these pathways, we filtered the data again based on time, and an ANOVA was performed to identify genes significantly altered based on exposure (saline or sarin) at each time point following seizure onset. When the data was analyzed at individual time points, we found that apoptosis signaling was significant at 6 h; induction of apoptosis by HIV1 was significant at 3 and 6 h; karatan sulfate biosynthesis was significant at 0.25, 3, and 6 h; and role of osteoblasts, osteoclasts, and chondrocytes in rheumatoid arthritis was not significant at any of the time points analyzed. The -log of the p-value are graphed for each time point (1.3 = -log of 0.05). [file 1742-2094-8-84-S9.PNG]

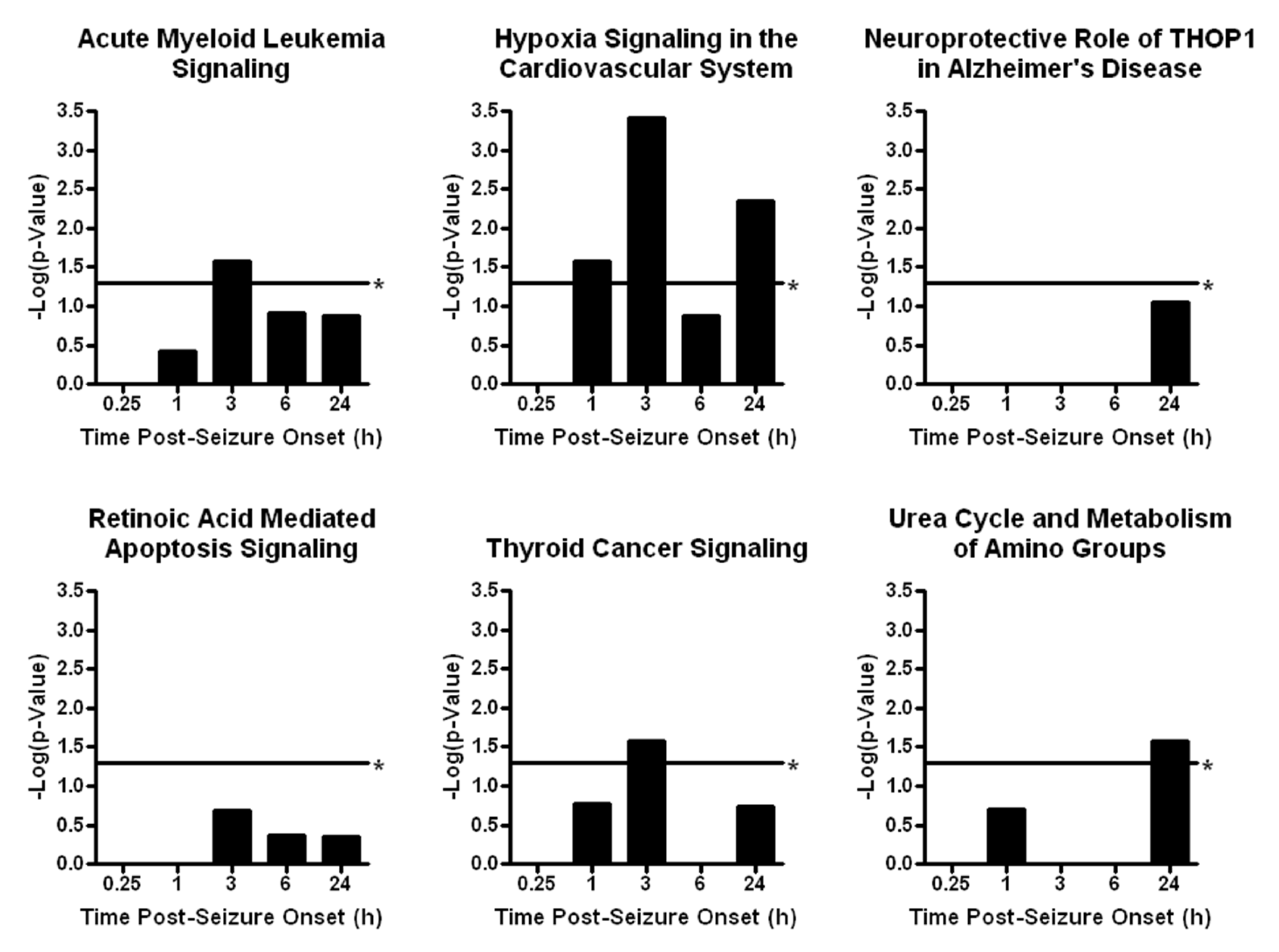

Supplement: Additional file 10 — Canonical pathways significantly altered only in the hippocampus of sarin-exposed seizing animals. When the data was analyzed at individual time points, we found that acute myeloid leukemia signaling and thyroid cancer signaling were significant at 3 h; urea cycle and metabolism of amino groups was significant at 24 h; and hypoxia signaling in the cardiovascular system was significant at 1, 3, and 24 h following seizure onset. Neuroprotective role of THOP1 in Alzheimer's disease and retinoic acid mediated apoptosis signaling were not significant at any of the individual time points examined. [file 1742-2094-8-84-S10.PNG]

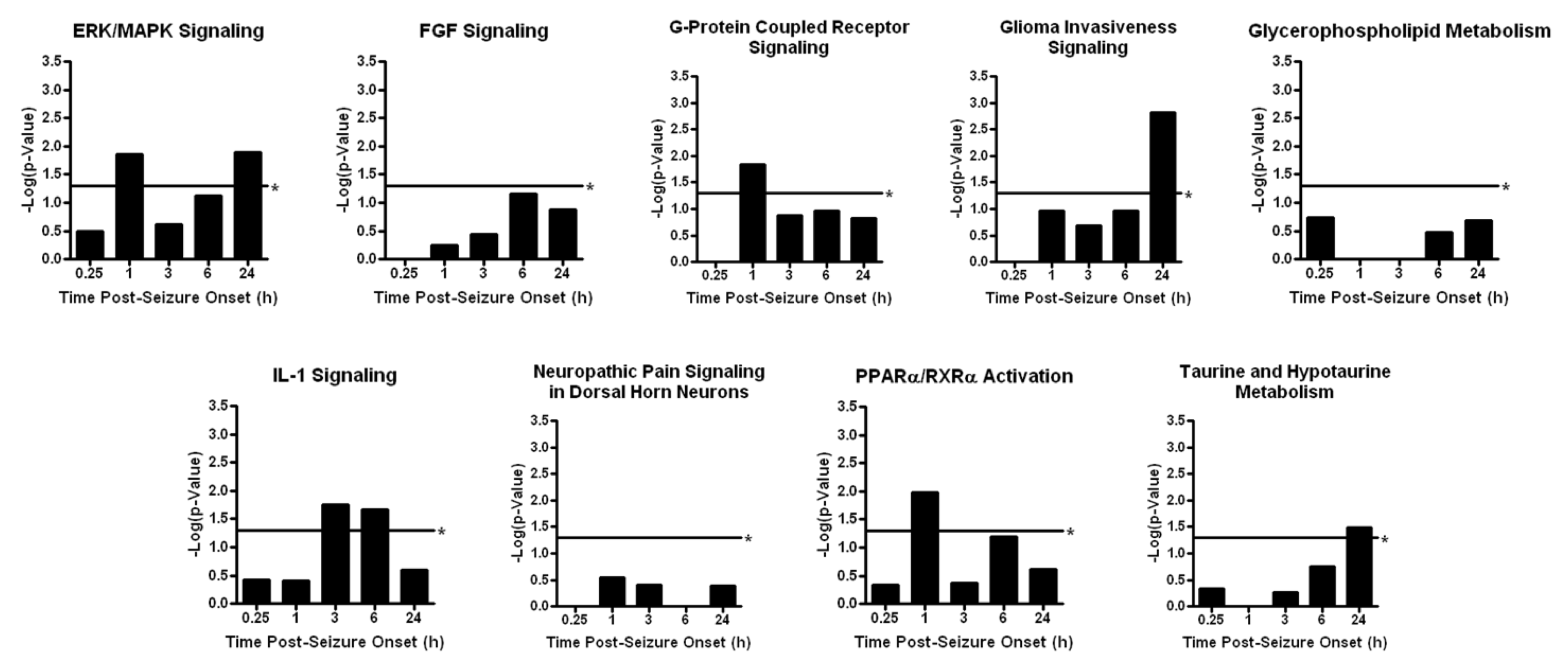

Supplement: Additional file 11 — Canonical pathways significantly altered only in the piriform cortex of sarin-exposed seizing animals. When analyzed at individual time points, we found that G-protein coupled receptor signaling and PPARα/RXRα activation were significant at 1 h; glioma invasiveness signaling and taurine and hypotaurine metabolism were significant at 24 h; ERK/MAPK signaling was significant at 1 and 24 h; and IL-1 signaling was significant at 3 and 6 h. FGF signaling, glycerophospholipid metabolism, and neuropathic pain signaling in dorsal horn neurons were not significant at any time point analyzed. [file 1742-2094-8-84-S11.PNG]

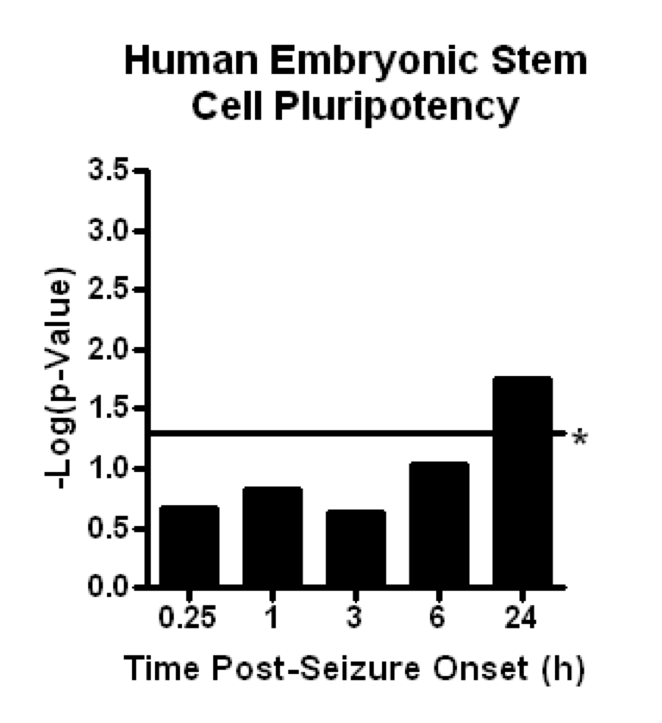

Supplement: Additional file 12 — Canonical pathway significantly altered only in the septum of sarin-exposed seizing animals. When analyzed at individual time points, it was significant only at 24 h after seizure onset. [file 1742-2094-8-84-S12.PNG]

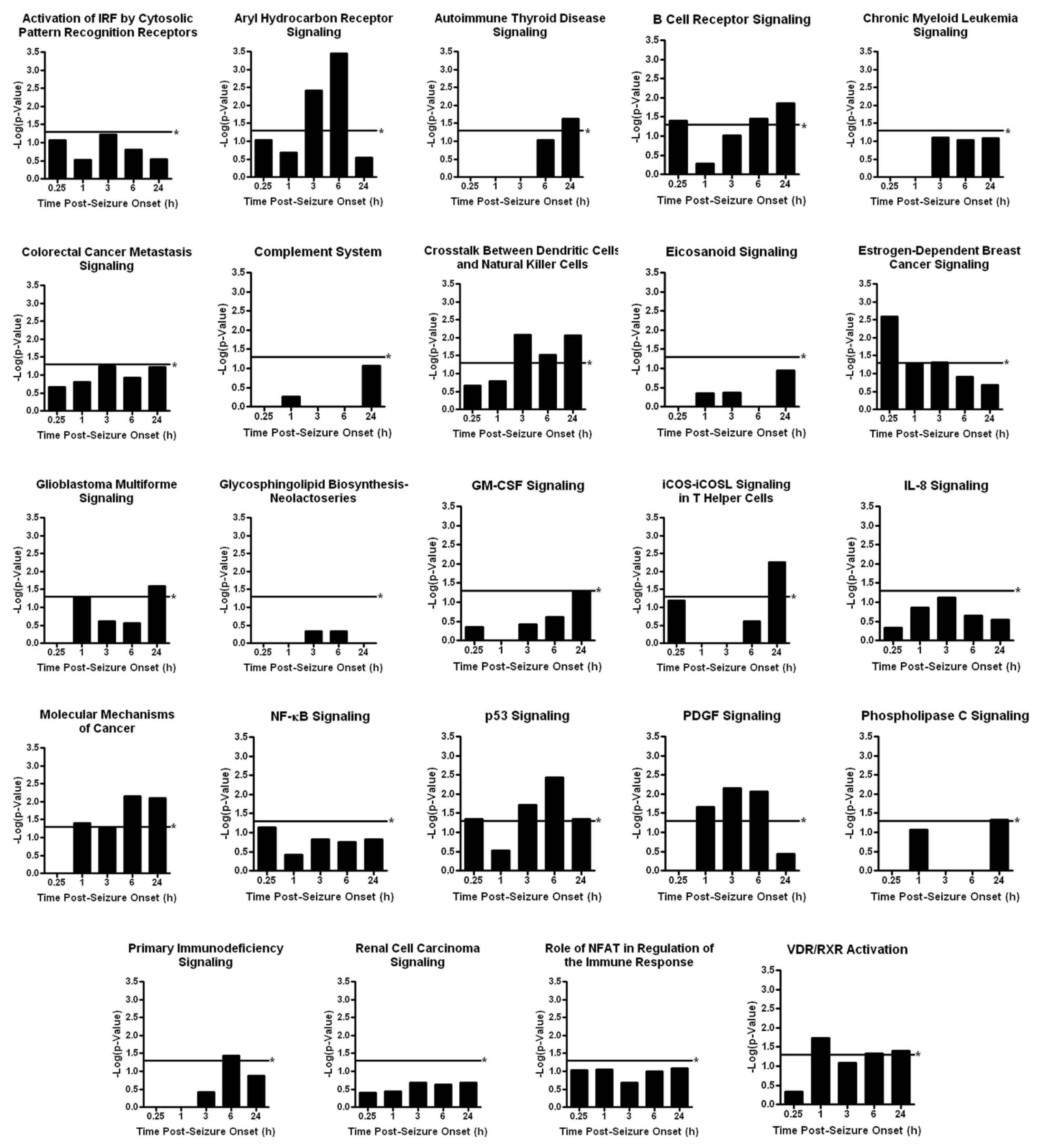

Supplement: Additional file 13 — Canonical pathways significantly altered only in the thalamus of sarin-exposed seizing animals. When the data was analyzed at individual time points, we found that primary immunodeficiency signaling was significant at 6 h; autoimmune thyroid disease, glioblastoma multiforme signaling, iCOS-iCOSL signaling in T helper cells, and phospholipase C signaling were significant at 24 h; estrogen-dependent breast cancer signaling was significant at 0.25 and 3 h; aryl hydrocarbon receptor signaling was significant at 3 and 6 h; B cell receptor signaling was significant at 0.25, 6, and 24 h; PDGF signaling was significant at 1, 3, and 6 h; VDR/RXR activation was significant at 1, 6, and 24 h; crosstalk between dendritic cells and natural killer cells was significant at 3, 6, and 24 h; p53 signaling was significant at 0.25, 3, 6, and 24 h; and molecular mechanisms of cancer was significant at 1, 3, 6, and 24 h after seizure onset. The remaining pathways were not significantly altered in the thalamus at any individual time point following sarin-induced seizure onset. [file 1742-2094-8-84-S13.PNG]

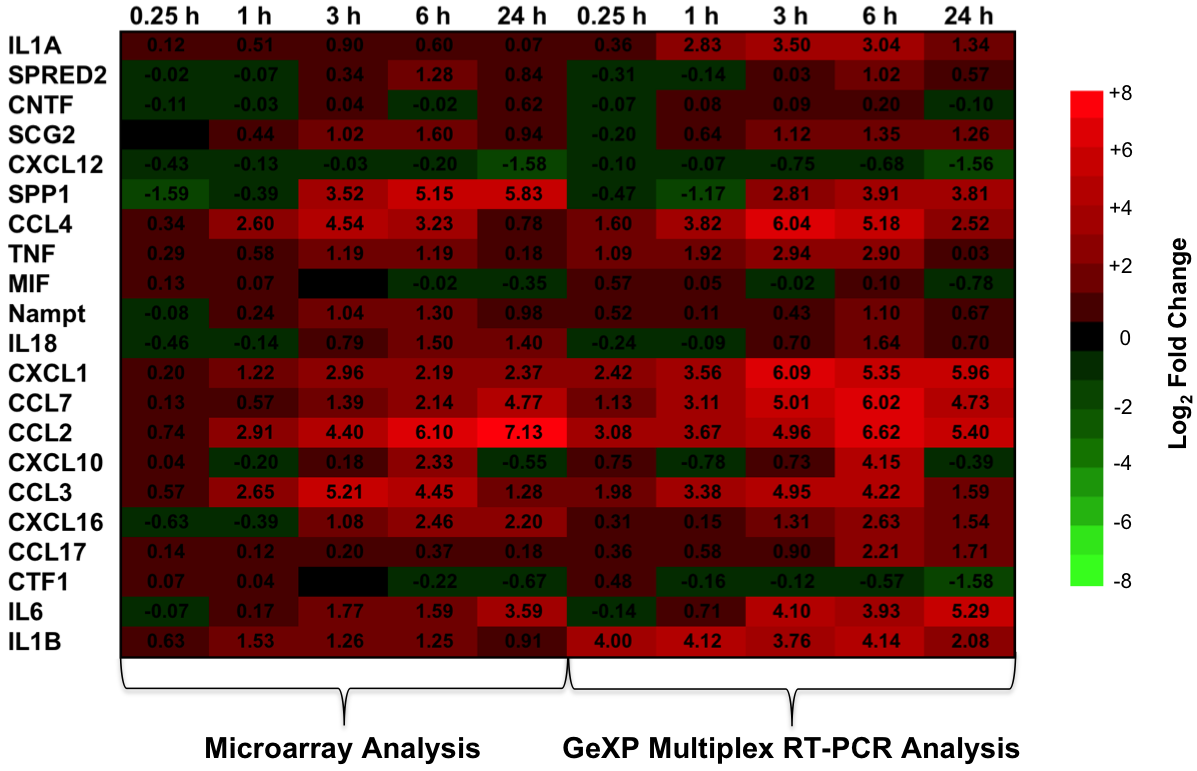

Supplement: Additional file 15 — Microarray analysis and multiplexed RT-PCR show similar gene expression changes in amygdala following sarin-induced seizure. The GeXP genetic analysis system was used to measure the expression levels of 21 differentially expressed cytokines or chemokines by multiplexed RT-PCR to validate the microarray data. The expression of each gene within a sample was normalized to GAPDH expression to minimize inter-capillary variation, and the normalized intensity of each replicate (n ≥ 3) was used to calculate an average intensity of each sample group (i.e. control or sarin-induced seizure at each time point). The fold expression difference between control and sarin-induced seizure samples is shown for each gene at each of the five time points examined. The fold changes in expression obtained in the microarray analysis are shown on the left, and the fold changes in expression obtained in the multiplex PCR analysis are shown on the right. Genes that were down-regulated following sarin-induced seizure are shaded in green, and genes that were up-regulated following sarin-induced seizure are shaded in red. Changes in expression were not collected for some time points using multiplex RT-PCR because transcript levels in the control samples were too low to be detected by the instrument (indicated by an asterisk). [file 1742-2094-8-84-S15.PNG]

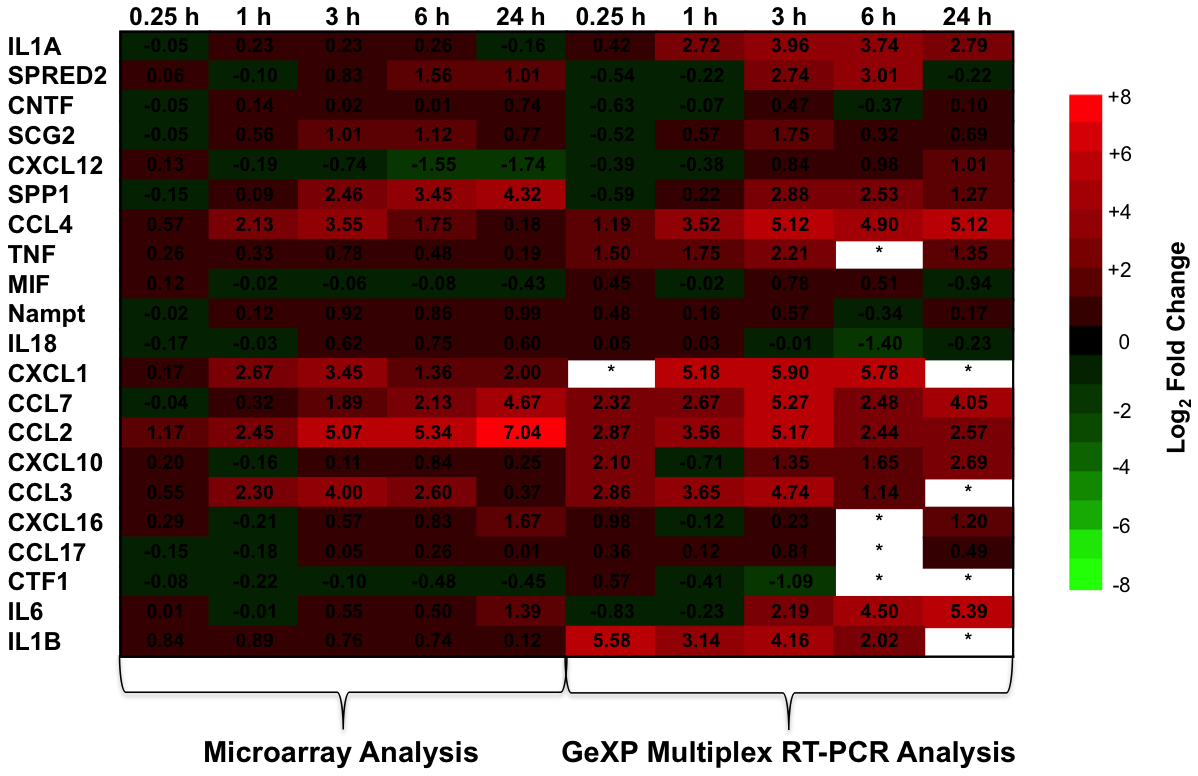

Supplement: Additional file 16 — Microarray analysis and multiplexed RT-PCR show similar gene expression changes in hippocampus following sarin-induced seizure. [file 1742-2094-8-84-S16.PNG]

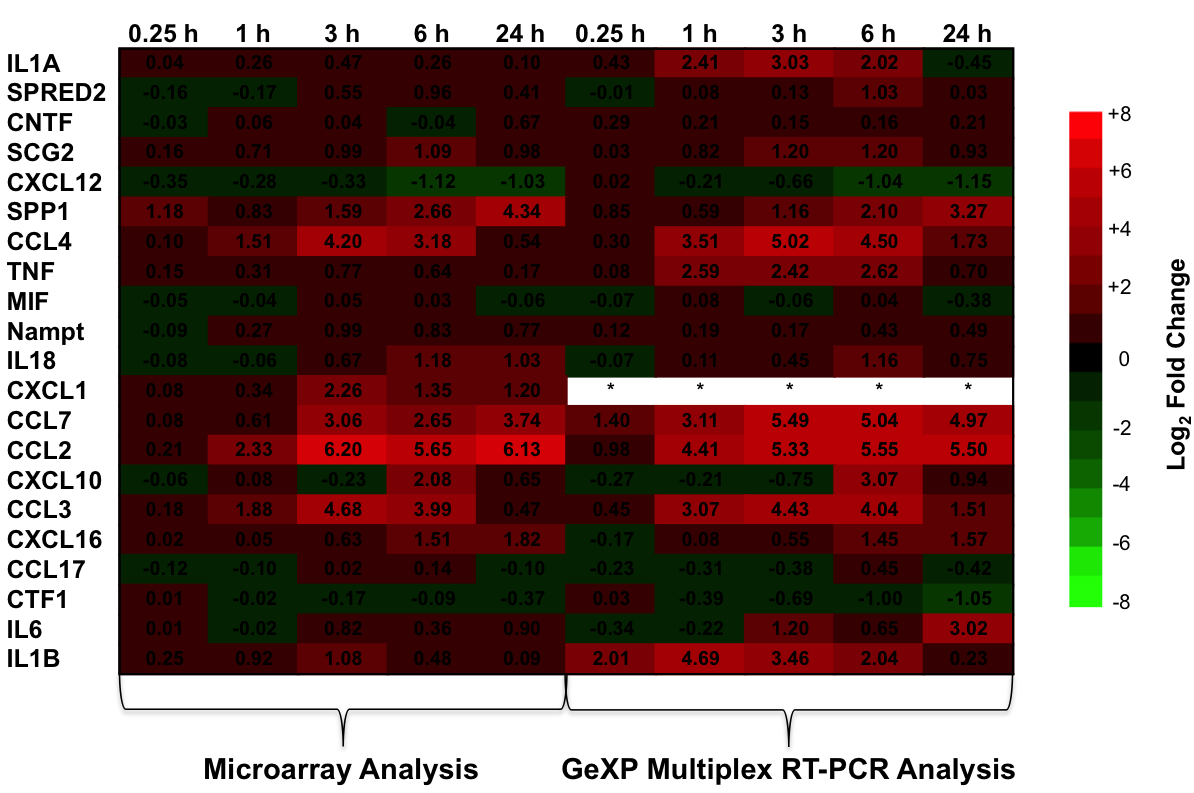

Supplement: Additional file 17 — Microarray analysis and multiplexed RT-PCR show similar gene expression changes in septum following sarin-induced seizure. [file 1742-2094-8-84-S17.PNG]

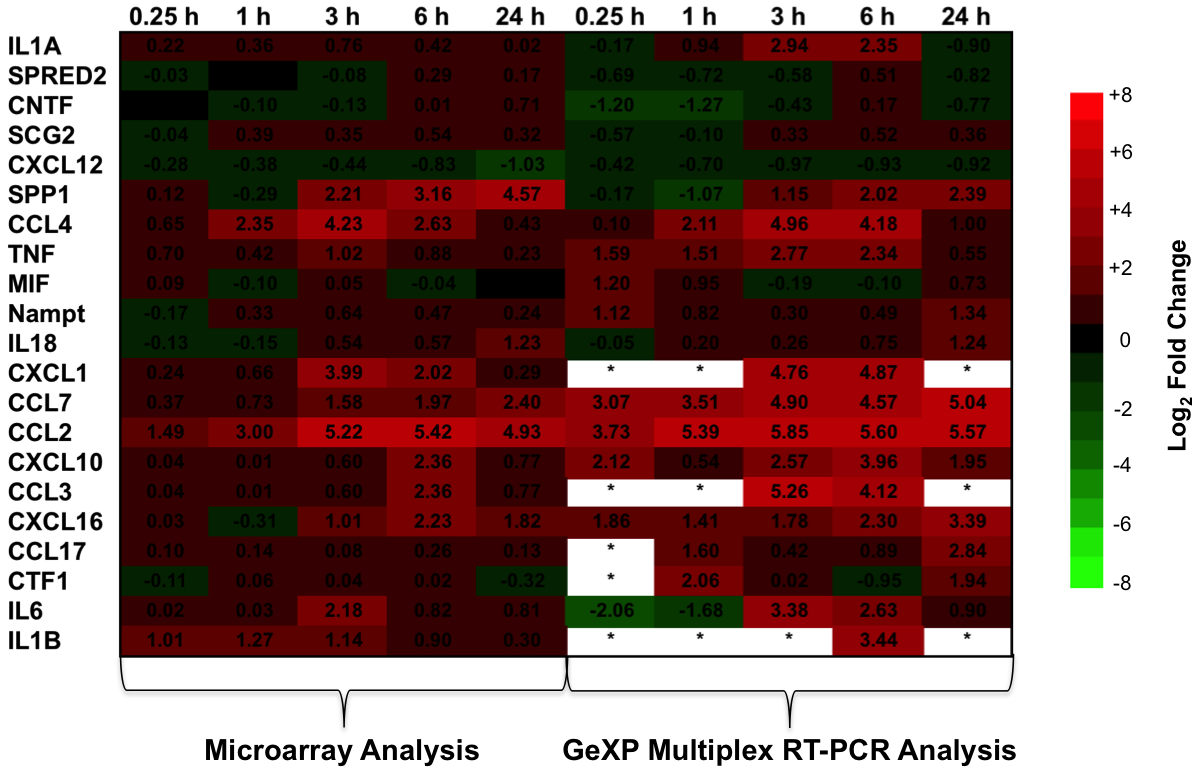

Supplement: Additional file 18 — Microarray analysis and multiplexed RT-PCR show similar gene expression changes in thalamus following sarin-induced seizure. [file 1742-2094-8-84-S18.PNG]
